# Silver nanoparticles-decorated Preyssler functionalized cellulose biocomposite as a novel and efficient catalyst for the synthesis of 2-amino-4*H*-pyrans and spirochromenes

**DOI:** 10.1038/s41598-020-70738-z

**Published:** 2020-09-03

**Authors:** Sara Saneinezhad, Leila Mohammadi, Vahideh Zadsirjan, Fatemeh F. Bamoharram, Majid M. Heravi

**Affiliations:** 1 Department of Chemistry, Mashhad Branch, Islamic Azad University, Mashhad, Iran; 2 Department of Chemistry, Alzahra University, Tehran, Iran

**Keywords:** Chemical biology, Catalysis

## Abstract

Silver nanoparticles-decorated Preyssler functionalized cellulose biocomposite (PC/AgNPs) was prepared and fully characterized by FTIR, UV–vis, SEM, and TEM techniques. The preparation of PC/AgNPs was studied systematically to optimize the processing parameters by Taguchi method using the amount of PC, reaction temperature, concentration of silver nitrate and pH of medium. Taguchi’s L9 orthogonal (4 parameters, 4 level) was used for the experimental design. The SEM analysis confirmed the presence of the Preyssler as a white cloud as well as spherical AgNPs on the surface of cellulose. The formation of AgNPs on the surface was observed by changing of the color from yellow to deep brown and confirmed by UV–vis spectroscopy. The best yield of AgNPs forming was obtained in pH 12.5 at 80 ºC in 20 min. TEM analysis confirmed the formation of spherical AgNPs with a size of 50 nm, at the 1% wt. loading of Preyssler. This easily prepared PC/AgNPs was successfully employed as an efficient, green, and reusable catalyst in the synthesis of a wide range of 2-amino-4*H*-pyran and functionalized spirochromene derivatives via a one-pot, multicomponent reaction. The chief merits realized for this protocol were the utilization of commercially available or easily accessible starting materials, operational simplicity, facile work-up procedure, obtaining of high to excellent yields of the products and being done under green conditions. The catalyst could be easily separated from the reaction mixture and reused several times without observing any appreciable loss in its efficiency.

## Introduction

With increasing concern about environmental pollution, the development of new heterogeneous catalytic systems based on green and biodegradable solid supports have attracted much attention of chemical community. In this area, renewable and biodegradable organic polymers, biopolymers, and especially polysaccharide-supported catalysts, with or without modification of the support, such as starch, cellulose^1^ and hemicellulose^2,3^, chitosan,^4,5^ guar-gum,^6^ carrageenans^7^ and lignin^8^ have been gaining increased attention in the last decade. Among these heterogeneous supports, cellulose as an inexpensive and biodegradable natural biopolymer has been proved to be an attractive candidate as supporting materials. It can be used as microcrystalline cellulose, cellulose hydrogel, cellulose membrane, and cellulose microsphere etc.^9–12^.

Literature survey shows among different heterogeneous catalytic reactions, many investigations have been devoted to develop methodologies based on the use of metal nanoparticles supported onto cellulose due to its specificity towards attracting metal ions^13–23^. Deposition of nanoparticles on the surface of biopolymers is a subject of great interest due to their potential applications in electronic devices, sensing, catalysis and bio-medical sciences. Along this line, cellulose-based catalysts coordinated metals, such as palladium, ruthenium, platinum, copper, silver, and nickel nanoparticles, have been prepared and used, continuously^24–29^. Direct preparation of metal nanoparticles on supports has attracted a considerable interest mainly due their potential applications in optics, electronic devices, catalysis, sensors, medical applications, etc.^18,30–34^. In all these reactions, metals have been tried being deposited on cellulose, directly. However due to instability of bonds between metals and cellulose, they could easily leach from cellulose after one or two reactions. To circumvent this problem, functionalization cellulose is used make cellulose-metal complexes more stable and efficient for being used as catalyst. Numerous examples of cellulose-supported organic transformations are reported in the literature^35–37^. For example, a catalytic system including Co(II) supported on ethylenediamine functionalized nanostructured cellulose has as an efficient and reusable catalyst for aerobic oxidation of various benzylalcohols has been reported by Shaabani et al.^38^.

Additionally, in the Suzuki reaction, Due et al. developed an air-stable diphenylphosphinate- modified cellulose-supported catalyst^39^, and Keshipour et al. used ethylenediamine for functionalization of cellulose^37^. Wang et al. attached triphenylphosphine and *N*-methylimidazole to cellulose to make as support of Pd and used it in Suzuki reaction^40^. Furthermore, thiol modified cellulose fibers–gold nanoparticles composites served as active catalysts for the reduction of 4-nitrophenol into 4-aminophenol^41^. Palladium supported on 2-aminopyridine functionalized cellulose was also synthesized and fully characterized by Peibo et al.^42^, and its catalytic potency and recyclability were examined successfully in one of the classic name reaction for the formation of carbon–carbon bond so-called, the Suzuki reaction. In spite of all these promising and motivating achievements, the immobilization of MNPs on the inorganic functionalized cellulosic substrates persisted being low thus, much attempt must made to develop for replacing organic functionalizing materials with conventional inorganic materials.

Using heteropolyacids (HPAs) as benign and environmentally green inorganic polymers, finally led to the fabrication of engineered and eco-friendly functionalized biopolymers. Preyssler heteropolyacid is a kind of heteropolyacids, which comprises a cyclic assemblage of five PW_6_O_22_ segments, each consequent of the spherical Keggin anion, [PW_12_O_40_]^3−^, through the exclusion of two groups of three corner-sharing trioxide tungsten octahedron. This structure contains five PO_4_ tetrahedron encircled by thirty trioxide tungsten, associated to each other by power and corner-sharing oxygens^43^. The intrinsic typical of Preyssler coordinated with metals strictly brands it as a prevalent ligand to back up different metals thus, could be used as the bridge between metals and the matrix to generate well-organized and effective catalysts^44^. Recently, we achieved an effective strategy for the designing and in situ synthesis of an organic–inorganic polymeric bio-composite involving functionalized microcrystalline cellulose and Preyssler HPA for immobilization of Pd nanoparticles on the surface to the formation of nanobiocomposite as surface heterogeneous composite and used it in successful degradation of azo dyes^45^.

**Figure 1:**
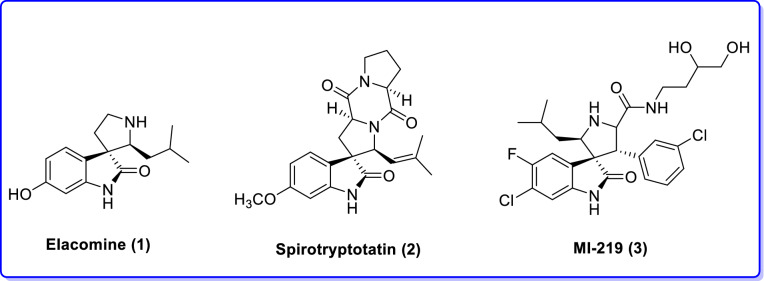
Spirooxindoles showing biological activities.

Nowadays, multicomponent reactions (MCRs) have attracted much attention both in academia and industry owing to their unique synthetic efficacy, inherent atom economy, high selectivity and operational simplicity^46^. Several divers and interesting heterocyclic systems, particularly those which are beneficial in combinatorial chemistry as powerful tools in drug discovery, have been conveniently synthesized via MCR^47^. MCRs are also expedient for the practical establishment of chemical libraries of structurally related, medicinally important drug-like compounds^48^. Thus, the design of new MCRs have attracted enormous attention particularly in the region of drug discovery and synthesis of complex molecules and natural products. MCRs particularly those conducted in water or aqueous medium, nowadays found delight and very high respect in synthetic organic chemistry for the preparation of chemically and biologically vital compounds by justification of their eco-friendly, instinct atom-economy and green features^49–54^. For these reasons, MCRs which are naturally being done in one-pot fashion can vividly reduce the creation of chemical waste and show high impact of the cost starting materials, shorten reaction times, and afford higher overall chemical yields^55–57^. Among heterocyclic systems, pyran and its derivatives are recognized as a significant class of compounds, which set up the important and abundant core in different naturally occurring important compounds as well as photochromic materials^58,59^. They also exhibit a broad range of biological potencies such as anticancer^59^, antimicrobial^60^, antioxidant^61^ and antiproliferative activities^62,63^. 4*H*-Pyran derivatives are also found being powerful calcium channel blockers, which are structurally comparable to potentially active 1,4-dihydropyridines^64^. Furthermore, 2-amino-4*H*-pyrans are frequently applied in cosmetics and pigments, or are also can be used as potentially biodegradable agrochemicals. The 4*H*-pyran derivatives containing a nitrile functional group are also valuable intermediates for the synthesis of a wide range of compounds for example, lactones, pyridones, aminopyrimidines, 1,4-dihydropyridines, pyranopyrazoles, and imidoesters^65,66^. Due to imperative above-mentioned properties of pyran derivatives, synthesis of this heterocyclic system stand in paramount of has gained of organic synthesis^67^. The most direct and frequently used synthetic approach for such heterocyclic system, comprises MCR (a three-component component reaction), including an aldehyde, various alkylmalonates and diverse enolizable C–H activated acidic compounds catalyzed by homogeneous or heterogeneous catalysts such as diammonium hydrogen phosphate^68^, N-methylimidazole^69^, 4-(dimethylamino)pyridine (DMAP)^70^, lithium bromide^71^, hexamethylenetetramine^72^, 1,8-diazabicyclo[5.4.0]-undec-7-ene (DBU)^73^, potassium phthalimide-*N*-oxyl^74^, lipase^75^, Ce(SO_4_)_2_·4H_2_O^76^, cerium(III) chloride^77^, tetrabutylammonium fluoride (TBAF)^78^, or a basic ionic liquid^79^. The latter is usually proceeds smoothly in glycerol^80^ or choline chloride-urea^81^, agro‐waste based Water Extract of Muskmelon Fruit Shell Ash (WEMFSA)^82^, and amine-functionalized SiO_2_@Fe_3_O_4_ nanoparticles^83^. On the other hand, spirooxindole derivatives are also striking objectives in synthetic organic chemistry by benefit of their highly obviously biological potencies as well as broad-ranging efficacy as intermediates in the total synthesis of alkaloids, drug candidates, and clinical medicines^84,85^. Additionally, it has been found that the presence of the indole 3-carbon atom in the construction of spiroindoline derivatives can extremely increase their biological potencies^78,86–90^. As examples, 3,3‐spirooxindole cores with inhibited potency have been illustrated in Fig. 1^91–93^. For these reasons, the synthesis of these compounds has stirred up the interest of synthetic organic chemists^94^. Accordingly, there have been a few reports^95^ concerning MCRs for the synthesis of spirooxindole derivatives in aqueous media, using a plethora of different catalyst, for example L-proline^96^, TEBA^97^, and NH_4_Cl^96^ have been employed in these reactions, enhancing the difficulty of purifications.

**Scheme 1:**
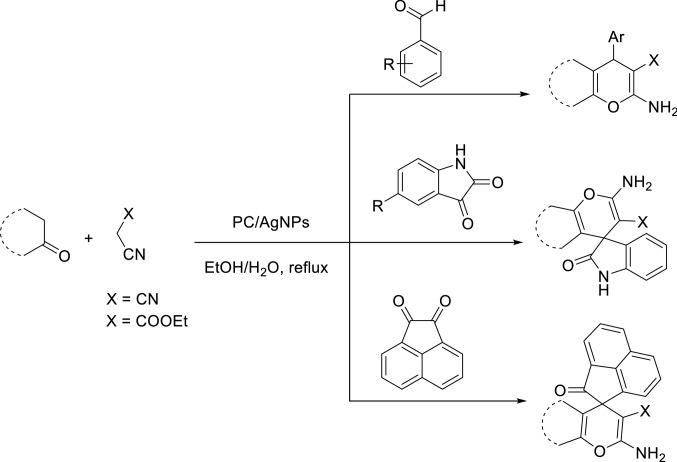
An efficient one-pot synthesis of functionalized 2-amino-4*H*-pyrans and spirochromenes in the presence of PC/AgNPs catalyst.

We are interested in heterocyclic chemistry^98–108^ especially in the synthesis of heterocycles via MCR^109,110^ being synthesized in the presence of heterogeneous catalysis in water^111–113^. In last few decades, our research group has focused on the heteopolyacids and their polyoxmetalates-catalyzed reactions. The results of these achievements have been collected in several review articles, useful to those synthetic organic chemists who are interested in HPAs-catalyzed reactions^114^. We have also recently reported the preparation and applications of immobilized AgNPs^115–120^. Based on the points mentioned above and in continuation of our interest in exploring green heterogeneous catalysts for organic transformations resulting in the construction of the heterocyclic systems^111,121,122^, herein we wish to report our successful attempt to apply our novel and fully characterized PC/AgNPs as an efficient and reusable catalyst in the synthesis of 4*H*-pyrans and spirochromenes via a one-pot three-component cyclocondensation reactions. A wide range of substrates including differently substituted benzaldehydes, isatin derivatives, and acenaphthenequinone are condensed with enolizable C-H activated compounds and alkylmalonates to afford a wide range of the desired products in good high yields (Scheme 1).

## Experimental

### Materials and methods

#### Materials

All chemicals were purchased from Merck and Sigma–Aldrich Company and utilized as received. Microcrystalline cellulose (Cotton linters powder), ethanol (99.5% w/w), nitric acid (65% w/w); ammonia solution (25% w/w), tetraethyl orthosilicate (98% w/w), 3- aminopropyltriethoxysilane (95% w/w), and silver chloride were purchased from Sigma–Aldrich Company and used as received. Preyssler heteropolyacid and functionalized cellulose-Preyssler biocomposites were prepared according to our earlier work^44,123^.

#### Instruments

The Scanning Electron Microscope (SEM) model VP-1450 (LEO, Co., Germany), was used for SEM analysis. For transmission electron microscopy (TEM) analysis, an LEO 912 AB instrument was used. The formation of AgNPs was studied by UV–vis spectra using Milton Roy, Spectvonic 1,001 plus spectrophotometer. Using the ATR method, infrared absorption spectra were recorded in KBr pellets on a VERTEX-70 infrared spectrometer.

Melting points were measured by an electro thermal 9,200 apparatus. IR spectra were recorded on a FT-IR Tensor 27 Spectrophotometer. All products were already known and identified by comparison of their physical (melting points) and spectral (FTIR spectra) data with those of authentic samples and found being identical.

#### Preparation of PC/AgNPs

In a typical reaction, 5 mL of AgNO_3_ solution (1.5 mM) and PC (1.0 g in 10 mL H_2_O), as a catalyst, were stirred at 50–80 °C, during vigorous stirring, hydrochloric acid or sodium hydroxide was added dropwise into the reaction mixture to adjust an appropriate pHs. After 5 min. silver nanoparticles (AgNPs) were precipitated as brown particles at pH = 6–12.5. At equal time intervals, the solutions were analyzed by a UV–vis spectroscopy to monitor the progress of the reaction.

#### Experimental design by Taguchi method

For experiential design, the Taguchi orthogonal array was applied. All parameters that had important impact on the preparation of AgNPs were selected as well as their levels. The amount of silver nitrate, temperature (°C), the amount of PC and pH were chosen as parameters at four levels. The concentration of silver nitrate was varied from 1.5–6 mM, the synthesis temperature was varied from 50 to 80 °C, the amount of PC was selected 0.13, 0.26, 0.52, 1 gr and the pH adjusted was between 5.5–12.5. Table 1 summarizes all of the parameters and levels used in this experiment.

**Table 1:** Parameters and levels used in the experiments using Taguchi robust design method with L_9_ orthogonal array.

Symbol	Parameters	Levels			
		1	2	3	4
A	pH	5.5	8.5	10.5	12.5
B	Loading PC (gr)	0.13	0.26	0.52	1
C	Temperature (°C)	50	60	70	80
D	Concentration of AgNO_3_ (mM)	1.5	3	4.5	6

##### Synthesis of 2-amino-4*H*-pyrans: general procedure

A mixture of an appropriate benzaldehyde (1 mmol), malononitrile/ethyl cyanoacetate (1 mmol) various 1,3-diketones (dimedone, barbituric acid or ethyl acetoacetate) or 4-hydroxycoumarin/3-methyl-1*H*-pyrazol-5(4*H*)-one, various β-dicarbonyl compounds (1,3-diketones (dimedone, barbituric acid or ethyl acetoacetate) or 4-hydroxycoumarin/3-methyl-1*H*-pyrazol-5(4*H*)-one mediated by PC/AgNPs (mg 0.025) was refluxed in EtOH/H_2_O (1:1, 5 ml) for the indicated reaction time in Tables 4, 5, 6, 7, and 8. The progress of the reaction was monitored by TLC (7:3 *n*-hexance/ethylacetate). Upon completion of the reaction (indicated by TLC), the mixture was filtered off under reduced pressure. The filtrate was cooled to room temperature and the precipitated solid was separated by filtration under reduced pressure. The respective product was pure enough but was further purified by crystallization from a mixture of EtOH/H_2_O to afford the respective desired product. The products were identified by comparison of their physical properties (melting points) as well as their FTIR spectral with those of already reported authentic compounds and found being identical.

##### Synthesis of spiro-2-amino-4*H*-pryans: general procedure

A mixture of a isatins or acenaphthenequinone (1 mmol), malononitrile or ethylcyanoacetate (1 mmol) and 1,3-diketones (dimedone, barbituric acid ethylacetoacetate) or 4-hydroxycoumarin/3-methyl-1*H*-pyrazol-5(4*H*)-one/α-naphtol or β-naphtol (1 mmol) was stirred in the presence of PC/AgNPs (mg 0.025) as a heterogeneous catalyst in H_2_O/EtOH EtOH/H_2_O (1:1, 5 mL) under reflux condition for an appropriate time as indicated in Tables 9 and 10. The progress of reaction was monitored by TLC (*n*-hexane/ethyl acetate (2:1). After completion of the reaction, the mixture was cooled to ambient temperature and the catalyst was separated by simple filtration. The filtrate was evaporated under reduced pressure and the obtained residue was crystalized from ethanol/*n*-hexane.

## Results and discussion

The interrelationship between the existed parameters for synthesis of nanoparticles to optimize the factors is a time and labor consuming work. Therefore, using statistical experimental design and the Taguchi method, in particular, have been performed by many researchers. Taguchi method can determine the experimental conditions having the least variability as the optimum condition. Not only, it is economical for characterizing a complicated process, but also it uses fewer experiments required in order to study all levels of all input parameters.

In this research, we performed statistical experimental design and Taguchi’s robust design concurrently. The statistical experimental design can be well-thought-out as the fresh data analysis, and the Taguchi’s robust design can be considered as the signal to noise (S/N) data analysis. The changeability can be conveyed by signal to noise (S/N) ratio.

**Figure 2:**
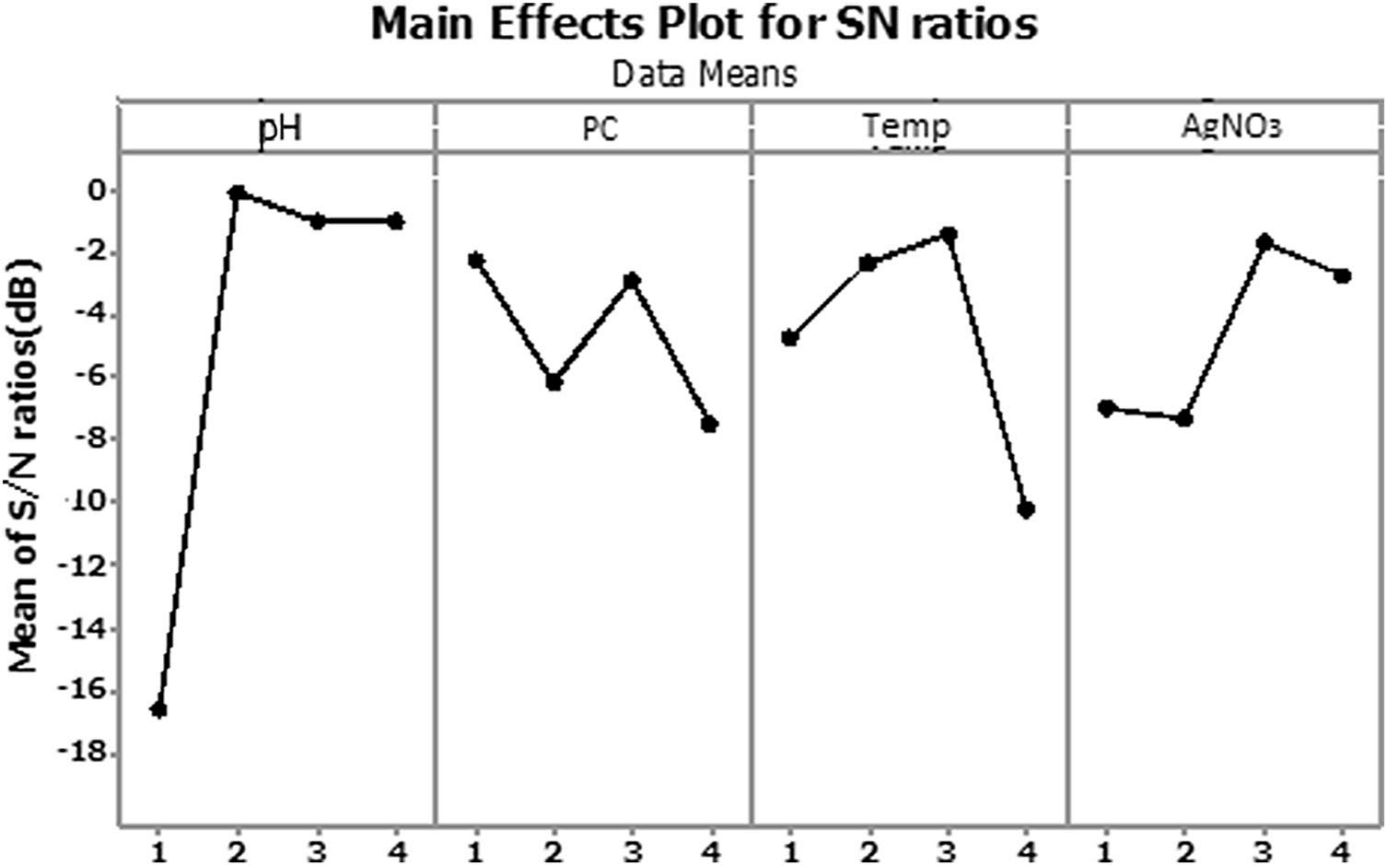
Main effect plot of AgNPs synthesized.

The effects of silver nitrate concentration, temperature, pH and different loadings PC on the UV–Vis analysis of silver nanoparticles at four different levels (1, 2, 3 and 4) were investigated in Fig. 2 and “plot” is a verb in this context.

Control factors are those design and process parameters that can be controlled in Taguchi method. Noise factors cannot be controlled during production or product use, but can be controlled during experimentation. In a Taguchi designed experiment, we manipulate noise factors to force variability to occur and from the results, identify optimal control factor settings that make the process or product robust, or resistant to variation from the noise factors. Higher values of the signal-to-noise ratio (S/N) identify control factor settings that minimize the effects of the noise factors. Main effects plot in Fig. 2 shows how each factor affects the response characteristic. A main effect exists when different levels of a factor affect the characteristic differently. In Fig. 2, the main effects plot for S/N ratio indicates that pH has the largest effect on the signal-to-noise ratio and after that, temperature shows the largest effect. The amount of PC and AgNO_3_ had the same effect on the signal-to-noise ratio.

Table 2 in the experimental section, shows the structure of Taguchi orthogonal robust design as well as the mean of S/N ratio for each level along with the UV–vis analysis measurements. In Fig. 2, higher values of the signal-to-noise response variable of one level, indicates a higher utility of that level than the other levels.

**Table 2:** Experimental measured signal to noise (S/N) ratios for Ag NPs.

xp.no	A	B	C	D	Absorbance	S/N ratio (dB)
1	1	1	1	1	0.24	− 12.39
2	1	2	2	2	0.13	− 17.72
3	1	3	3	3	0.28	− 11.05
4	1	4	4	4	0.054	− 25.35
5	2	1	2	3	1.86	5.39
6	2	2	1	4	0.78	− 2.15
7	2	3	4	1	0.53	− 5.51
8	2	4	3	2	1.25	1.93
9	3	1	3	4	2.34	7.38
10	3	2	4	3	0.91	− 0.81
11	3	3	1	2	0.61	− 4.29
12	3	4	2	1	0.49	− 6.19
13	4	1	4	2	0.35	− 9.11
14	4	2	3	1	0.64	− 3.87
15	4	3	2	4	2.94	9.36
16	4	4	1	3	0.98	− 0.17

### UV–visible results

With Taguchi experimental conditions (Table 2), including in situ one-pot synthesis of AgNPs without additional reducing agents was checked, showing a promising green catalyst with different loading of preyssler to prepare a new nanobiocomposite to catalyze the synthesis of 4*H*-pyrans. The formation of AgNPs on the surface of PC was observed by color change from white to deep brown in solution and confirmed by UV–vis spectroscopy.

**Figure 3:**
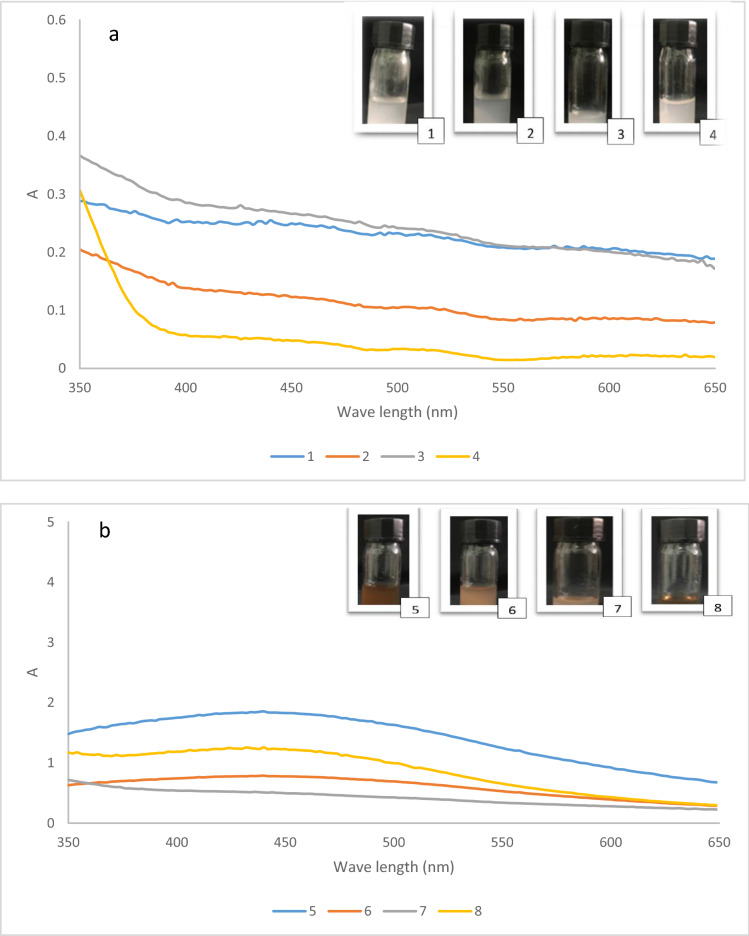

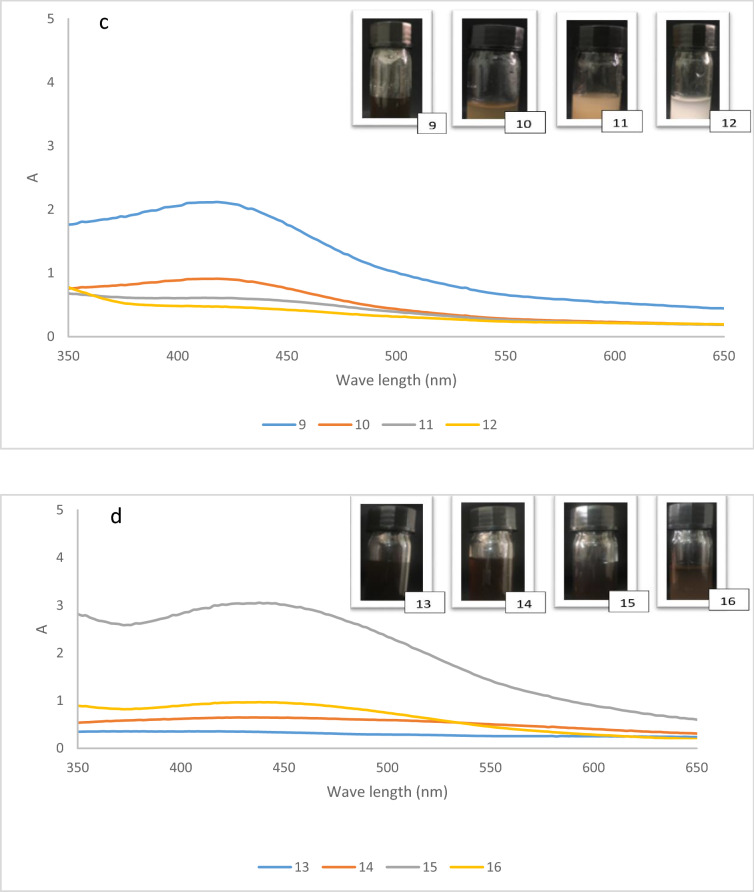
UV–vis spectra of AgNPs formation (**a**) pH = 5.5, (**b**) pH = 8.5, (**c**) pH = 10.5, (**d**) pH = 12.5.

Figure 3 exhibits the UV–vis spectra of AgNPs development showing a role of pH.

The absorption band at 440 nm is appeared as the result of formation of silver nanoparticles. The increase of the absorption band alongside with an optical color variation clearly showed the Ag^+^ ions were reduced to Ag (0). Ag^+^ can fully be subjected to reduction mediated by biocomposite PC and as a result a nanobiocomposite created in brown color at the bottom of the reaction pot. As it can be realized, the generation of AgNPs is related to the pH of the solution, so the best pH for preparation of AgNPs is obtained 12.5 in loading of 1 gr and AgNO_3_ 6 mM at 70–80 °C. The Preyssler anion make available extremely dispersed charged surfaces, that is perfect for being bounded to the metal ions through their aqueous predecessor solutions. These results suggest that Preyssler acts as reductant and stabilizer, which can lead to releasing the Preyssler in the solution and stabilizing of the AgNPs. This phenomenon strongly inhibited the deposition of AgNPs on the surface of the cellulose biocomposite. This is a common occurrence in the polyoxometalate catalysis when immobilized on different supports.

### SEM analysis

**Figure 4:**
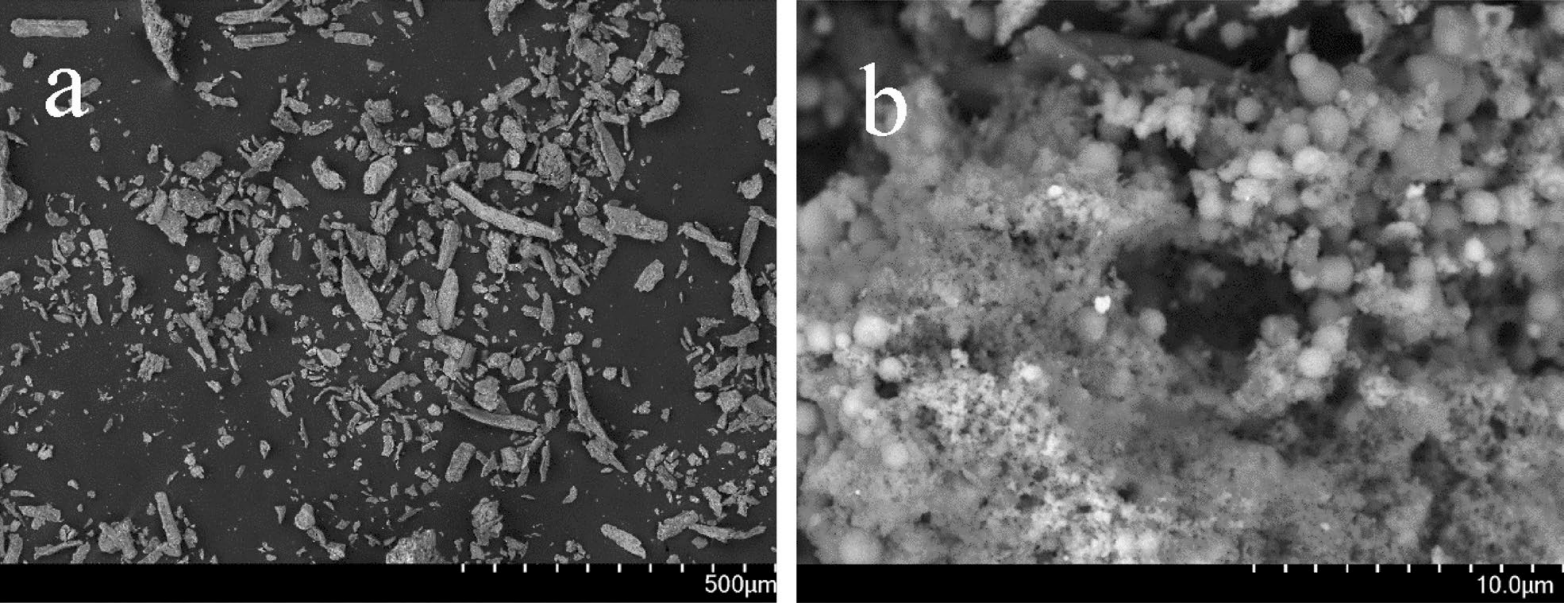
SEM images of (**a**) MCC and (**b**) PC /AgNPs (pH = 12.5, PC = 1 gr, temperature = 80 °C, AgNO_3_ = 6 mM).

Figure 4 shows the SEM images of Microcrystalline Cellulose (MCC) and PC /AgNPs. Figure 4b shows Preyssler were highly dispersed on MCC as a white cloud and excellent spherical architectures of the AgNPs. As shown in Fig. 4b, it is easy to distinguish between the PC and silver nanoparticles because of their different external morphology. It is suggested that the large interaction of Preyssler with functionalized cellulose could cause robust immobilization on cellulose with high surface density. On the other hand, the Preyssler anion provides highly distributed charged surfaces, which is ideal for binding the metal ions from their aqueous precursor solutions.

### TEM analysis

**Figure 5:**
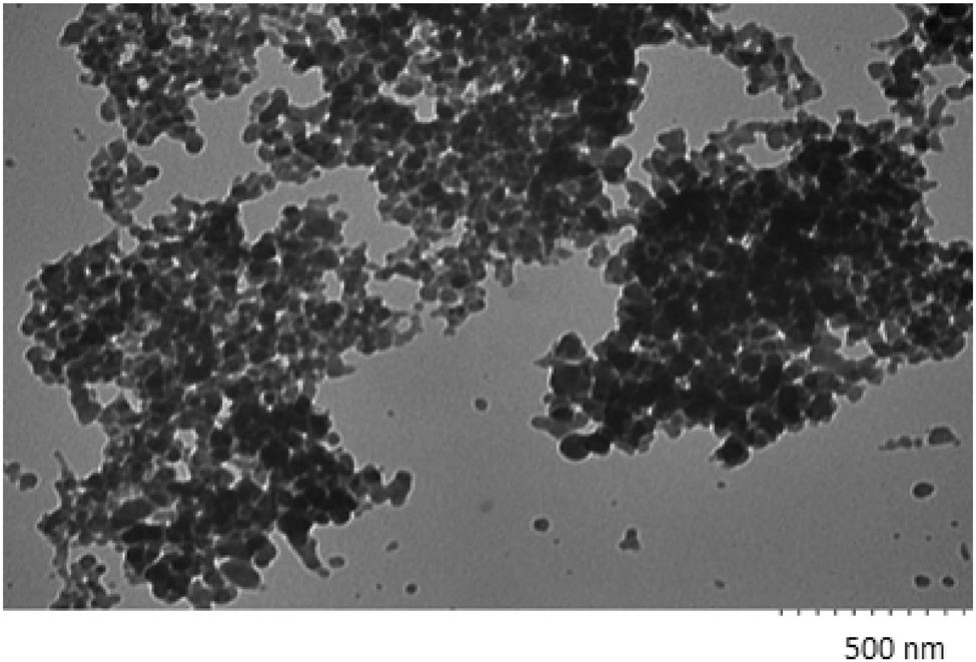
TEM images of AgNPs.

The TEM image of the AgNPs (Fig. 5) created by PC nanobiocomposite shows that the AgNPs are spherical in shape and circa 50 nm diameter.

### FTIR results

**Figure 6:**
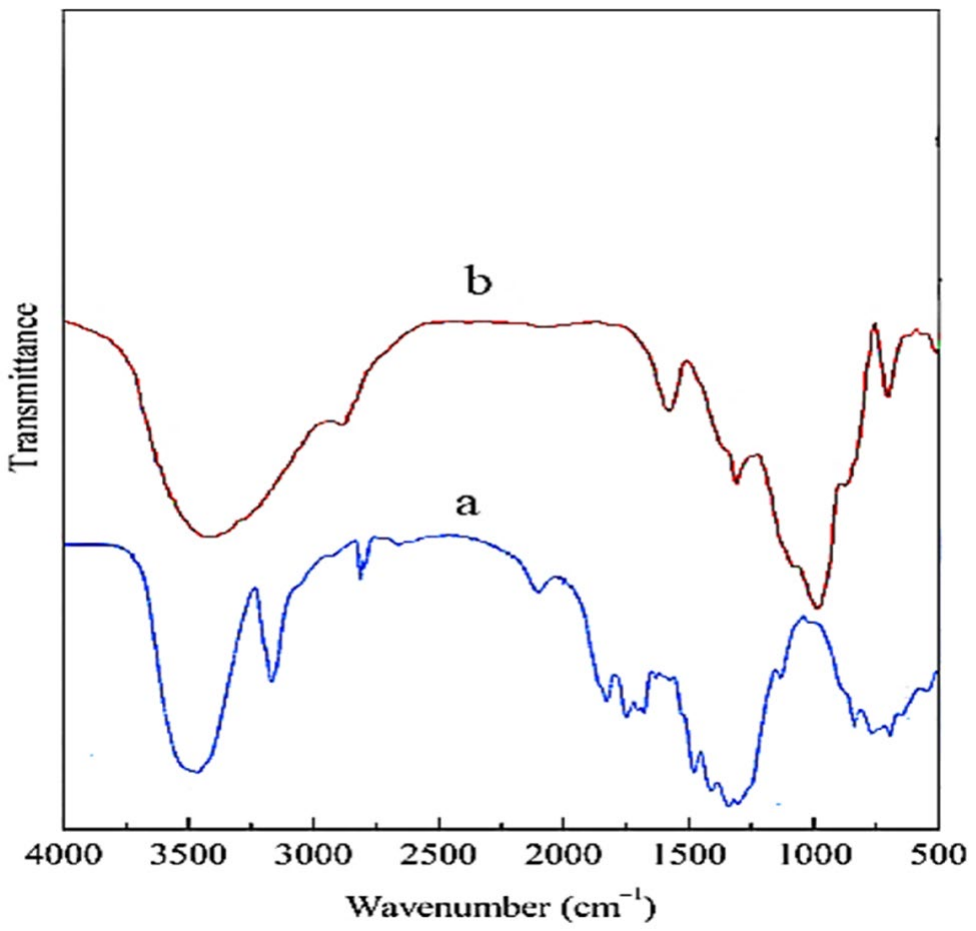
FTIR spectra of (**a**) MCC, (**b**) PC.

Figure 6a shows the FT-IR spectrum of the microcrystalline cellulose. A strong band at approximately 3,500 cm^−1^, is related to the stretching vibration of O–H groups. The characteristic peak around 2,800 cm^−1^ is attributed to the symmetric C–H vibrations. An adsorption band around 1,700 cm^−1^ is due to the absorbed water. Additionally, the peaks at around 1,200, and 670 cm^−1^ are related to the stretching vibration intermolecular ester bonding, and C–OH out-of-plane bending mode, respectively.

The existence of the Preyssler on the surface was confirmed by Fig. 6b, as we can see the existed peaks corresponds to Preyssler. Preyssler’s contains four kinds of oxygen that are in charge for the bands appeared in fingerprint region (between 1,200 and 600 cm^−1^) in the IR spectrum of Preyssler anion. The distinctive bands of the Preyssler anion, [NaP_5_W_30_O_110_]^14−^ are three different bands at 1,163 cm^−1^, 1,079 cm^−1^, and 1,022 cm^−1^, due to P-O stretching, respectively. In addition, two other bands at 941 cm^−1^ and 913 cm^−1^ can be ascribed to W–O–W a band at 757 cm^−1^ is attributed to W=O and a band at 536 cm^−1^ can be assigned to P-O bending. Interestingly, the above-mentioned bands can be weakly, strongly shifted, or oven covered in changed conditions. In the spectrum of PC, an addition to band appeared at 788 cm^−1^, assigned to (Si–O–Si), the band expected at about 1,080 cm^−1^ (Si–O–Si) was overlapped with the bands of cellulose in the same spectral region. In addition, the sharp band at 1,480 cm^−1^ is due to nitrate anions. Most of the absorption bands of Preyssler HPA were masked by functionalized cellulose matrix in 600–1,200 cm^−1^.

After definite determination structure of the PC/AgNPs catalyst, it was tested as heterogeneous nanocatalyst for the preparation of 2-amino-4*H*-pyrans through MCR in under green conditions (Table 3).

**Table 3:** Optimization of reaction conditions for the synthesis of 7-amino-5-(4-chlorophenyl)-2,4-dioxo-1,3,4,5-tetrahydro-2*H*-pyrano[2,3-*d*] pyrimidine-6-carbonitrile (**4b**) via MCR.
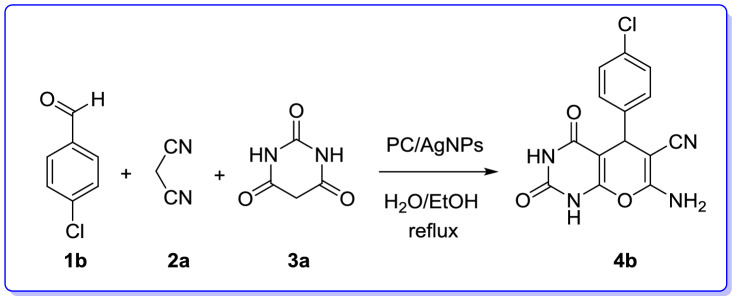

Entry	Solvent	Temperature	Catalyst amount (mol%)	Time (min)	Yield (%)
1	DMF	Reflux	0.025	180	70
2	CH_3_CN	Reflux	0.025	100	65
3	toluene	Reflux	0.025	110	60
4	CH_2_Cl_2_	Reflux	0.025	180	45
5	H_2_O	Reflux	0.025	120	40
6	EtOH	Reflux	0.025	120	60
7	EtOH/H_2_O	Reflux	–	420	Trace
8	EtOH/H_2_O	Reflux	0.025	20	95
9	EtOH/H_2_O	r.t	0.025	120	85
10	EtOH/H_2_O	Reflux	0.01	90	70
11	EtOH/H_2_O	Reflux	0.05	60	95
12	EtOH/H_2_O	Reflux	0.05	90	95

To find the secured optimal reaction conditions, the consequence of various factors such as catalyst loading, kind of solvent and reaction temperature were examined in a model reaction comprising 4-chlorobenzaldehyde, malononitrile and barbituric acid (Table 3). Among an unalike solvent the mixture of EtOH/H_2_O (1:1, 5 mL) the above reaction was found being proceeded more smoothly, completed in shorter time and giving better yield. As outlined in Table 3, for examining the influence of the catalyst on the progress of the reaction, the above mentioned model reaction was performed in the absence of the PC/AgNPs catalyst under reflux condition in a mixture of EtOH/H_2_O (1:1, 5 mL). As a result, only a trace amount of product was detected (Table 3, Entry 7). Furthermore, the effect of temperature was examined and the best transformation was observed when the reaction was conducted in refluxing EtOH/H_2_O (1:1, 5 mL) (Table 3, Entry 8). Besides, different solvents such as EtOH, toluene, DMF, CH_3_CN, EtOH/H_2_O and H_2_O were also tested (Table 3, entries 1–12). Our study disclosed that EtOH/H_2_O was the solvent of choice for this MCR (Table 3, Entry 9). The reaction was also performed employing different amounts of the catalyst involving 0.01, 0.025, 0.05 gr (Table 3, entries 10–12) and the results revealed that 0.025 gr of catalyst PC/AgNPs was optimal quantity of catalyst in refluxing EtOH/H_2_O (Table 3, entries 9).

Accordingly, the best result was obtained by employing of 0.025 gr of PC/AgNPs as catalyst in refluxing EtOH/H_2_O. Relied on the optimized reaction conditions, the catalytic activity of PC/AgNPs was examined in the synthesis of various 7-amino-tetrahydro-2*H*-pyrano[2,3-*d*] pyrimidines (**4a–j**) via the three-component reaction of substituted aromatic aldehydes, malononitrile or ethylcyanoacetate and barbituric acid. Differently substituted benzaldehydes involving electron-releasing groups such as (4-methoxybenzaldehyde, 4-methyl benzaldehyde) and electron-with drawing groups such as (4-nitrobenzaldehyde) were utilized successfully in this protocol to provide the desired products (**4a–j**) in satisfactory yields. In addition, when ethylcyanoacetate was utilized instead of malononitrile the corresponding 2-amino-4*H*-pyrans (**4f.-4j**) were obtained. Delightfully, the expected products were obtained in good to excellent yields as exhibited in Table 4.

**Table 4:** Synthesis of pyrano[2,3-*d*] pyrimidine derivatives in the presence of PC/AgNPs as catalyst via MCR.
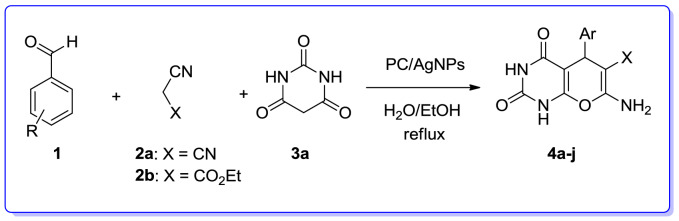

Entry	Ar	X	Time (min)	Product	Yield (%)	m.p. (ºC) Obs	m.p. (ºC) Lit
1	C_6_H_5_	CN	25	**4a**	91	222–224	221–224^124^
2	4-Cl–C_6_H_4_	CN	20	**4b**	96	262–264	263–265^80^
3	4-OMe–C_6_H_4_	CN	18	**4c**	95	267–269	266–270^125^
4	4-NO_2_–C_6_H_4_	CN	13	**4d**	90	238–240	238–240^126^
5	4-Me–C_6_H_4_	CN	15	**4e**	91	227–228	226–227^127^
6	4-Me–C_6_H_4_	CO_2_Et	18	**4f**	86	224–226	225^128^
7	4-OMe–C_6_H_4_	CO_2_Et	15	**4g**	85	298–299	297–298^80^
8	4-NO_2_–C_6_H_4_	CO_2_Et	15	**4h**	80	290–292	289–293^129^
9	4-Cl–C_6_H_4_	CO_2_Et	13	**4i**	95	> 300	> 300^80^
10	C_6_H_5_	CO_2_Et	25	**4j**	83	208–210	206–210^129^

In order to extend the substrate scope of this approach, we employed dimedone instead of barbituric acid. Three-component reaction of substituted aromatic aldehydes, malononitrile or ethylcyanoacetate and dimedone mediated by PC/AgNPs refluxing EtOH/H_2_O was successfully giving the expected corresponding products in satisfactory yields. The results are underlined in Table 5. As exhibited in the aforementioned table, the reaction of both electron-releasing on benzaldehydes such as 4-methylbenzaldehyde, 2-methoxybenzaldehyde, 3-methoxybenzaldehyde and 4-methoxybenzaldehyde and electron-withdrawing group on bezaldehydes such as 4-nitrobenzaldehyde, 4-chlorobenzaldehyde, 4-hydroxybenzaldehyde proceeded smoothly resulting in the construction of the corresponding products, substituted 2-amino-7,7-dimethyl-5-oxotetrahydro-4*H*-chromenes (**5a–n**) in satisfactory yield in relatively short reaction times.

**Table 5:** Three-component reaction for the preparation of 2-amino-7,7-dimethyl-5-oxotetrahydro-4*H*-chromenes (**5a**–**n**) in the presence of PC/AgNPs.
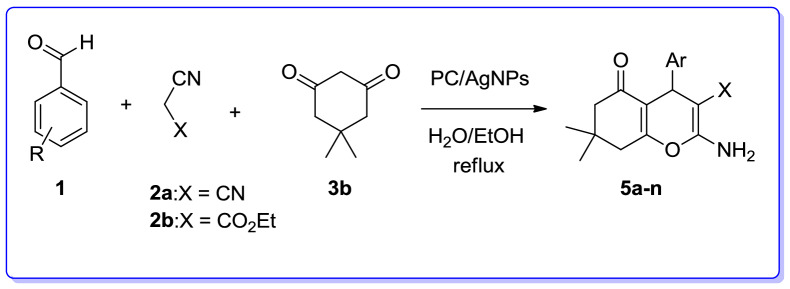

Entry	Ar	X	Time (min)	Product	Yield (%)	m.p. (ºC) Obs	m.p. (ºC) Lit
1	C_6_H_5_	CN	35	**5a**	82	222–224	222–224^76^
2	4-Cl–C_6_H_4_	CN	12	**5b**	96	212–214	213–215^80^
3	4-Me–C_6_H_4_	CN	18	**5c**	92	212–215	214–216^130^
4	4-OMe–C_6_H_4_	CN	13	**5d**	90	201–203	201–202^78^
5	2-OMe–C_6_H_4_	CN	13	**5e**	90	204–206	203–205^75^
6	3-OMe–C_6_H_4_	CN	14	**5f**	92	187–189	188–190^76^
7	4-NO_2_–C_6_H_4_	CN	12	**5g**	89	182–184	181–184^68^
8	4-OH–C_6_H_4_	CN	20	**5h**	85	211–212	210–212^131^
9	4-Me–C_6_H_4_	CO_2_Et	35	**5i**	78	154–156	156–157^132^
10	4-OMe–C_6_H_4_	CO_2_Et	30	**5j**	74	130–132	130–133^133^
11	4-NO_2_–C_6_H_4_	CO_2_Et	25	**5k**	70	180–182	181–183^133^
12	4-Cl–C_6_H_4_	CO_2_Et	20	**5l**	85	151–153	150–152^132^
13	3-NO_2_–C_6_H_4_	CO_2_Et	28	**5m**	81	155–156	156–157^74^
14	C_6_H_5_	CO_2_Et	50	**5n**	75	159–160	158–160^132^

Next, we examined the three-component reaction of different aromatic aldehydes, malononitrile or ethylcyanoacetate and 4-hydroxycoumarin in the presence of PC/AgNPs as an effective catalyst in EtOH/H_2_O under reflux condition. The 2-amino-5-oxo-4,5-dihydropyrano[3,2-*c*]chromenes were efficiently obtained in good to excellent yields (Table 6).

**Table 6:** Three-component reaction for the synthesis of 2-amino-5-oxo-4,5-dihydropyrano[3,2-*c*]chromenes (**6a**–**k**) in the presence of PC/AgNPs.
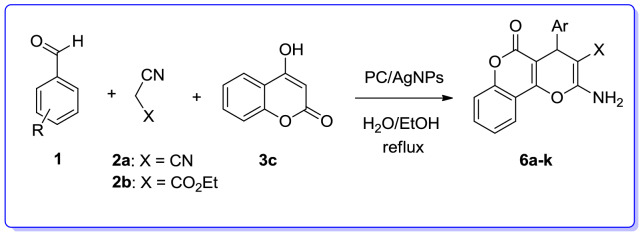

Entry	Ar	X	Time (min)	Product	Yield (%)	m.p. (ºC) Obs	m.p. (ºC) Lit
1	C_6_H_5_	CN	30	**6a**	80	254–256	256–257^73^
2	4-Cl–C_6_H_4_	CN	15	**6b**	89	265–266	264–266^70^
3	4-OMe–C_6_H_4_	CN	18	**6c**	83	239–240	238–240^73^
4	4-NO_2_–C_6_H_4_	CN	12	**6d**	80	251–253	252–254^79^
5	4-OH–C_6_H_4_	CN	25	**6e**	85	267–269	266–268^134^
6	4-Me–C_6_H_4_	CN	15	**6f**	82	254–257	255–256^135^
7	4-Me–C_6_H_4_	CO_2_Et	17	**6g**	75	113–115	114–117^136^
8	4-NO_2_–C_6_H_4_	CO_2_Et	14	**6h**	76	242–244	241–243^136^
9	4-Cl–C_6_H_4_	CO_2_Et	12	**6i**	74	193–195	192–194^136^
10	3-NO_2_–C_6_H_4_	CO_2_Et	20	**6j**	74	248–250	247–250^136^
11	C_6_H_5_	CO_2_Et	39	**6k**	73	188–190	187–189^136^

In order to establish the generality of this strategy, we conducted the three-component reaction of various substituted benzaldehydes, malononitrile/ethylcyanoacetate and 3-methyl-1*H*-pyrazol-5(4*H*)-one in the presence of PC/AgNPs in refluxing EtOH/H_2_O. As can be seen in Table 7, the desired products, 6-amino-3-methyl-1,4-dihydropyrano[2,3-*c*] pyrazoles (**7a–l**) were obtained in high yields.

**Table 7:** Three-component reaction for the preparation of 6-amino-3-methyl-1,4-dihydropyrano[2,3-*c*]pyrazoles (**7a**–**l**) catalyzed by PC/AgNPs.
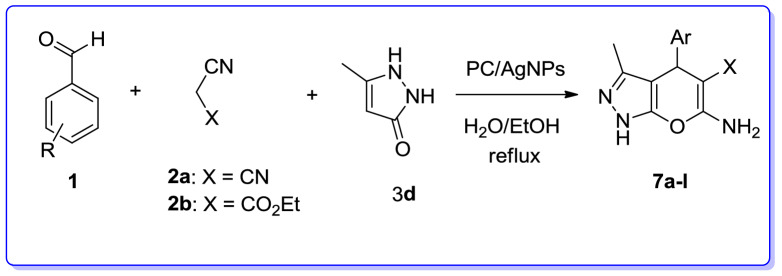

Entry	Ar	X	Time (min)	Product	Yield (%)	m.p. (ºC) Obs	m.p. (ºC) Lit
1	C_6_H_5_	CN	30	**7a**	86	244–246	244–246^137^
2	4-Cl–C_6_H_4_	CN	12	**7b**	95	231–233	232–234^138^
3	4-OMe–C_6_H_4_	CN	15	**7c**	94	173–175	174–175^139^
4	4-NO_2_–C_6_H_4_	CN	17	**7d**	96	196–198	195–196^139^
5	4-OH–C_6_H_4_	CN	25	**7e**	90	224–226	224–227^139^
6	4-Me–C_6_H_4_	CN	16	**7f**	89	214–216	215–217^140^
7	2-NO_2_–C_6_H_4_	CN	18	**7g**	89	220–222	220–222^141^
8	3-NO_2_–C_6_H_4_	CN	13	**7h**	91	193–195	193–195^142^
9	3-Br–C_6_H_4_	CN	25	**7i**	94	222–224	223–224^143^
10	3-NO_2_–C_6_H_4_	CO_2_Et	15	**7j**	92	179–182	180–182^144^
11	4-OCH_3_–C_6_H_4_	CO_2_Et	20	**7k**	85	163–166	166–168^145^
12	4-NO_2_–C_6_H_4_	CO_2_Et	25	**7l**	84	244–246	244–246^145^

The three-component reaction of various aromatic aldehydes, malononitrile and ethylacetoacetate was successfully accomplished in the presence of PC/AgNPs in refluxing EtOH/H_2_O to give ethyl 6-amino-5-cyano-2-methyl-4*H*-pyran-3-carboxylates (**8a–g**) in good yields (Table 8).

**Table 8:** Multicomponent reaction for the synthesis of ethyl 6-amino-5-cyano-2-methyl-4*H*-pyran-3-carboxylates (**8a**–**g**) catalyzed by PC/AgNPs.
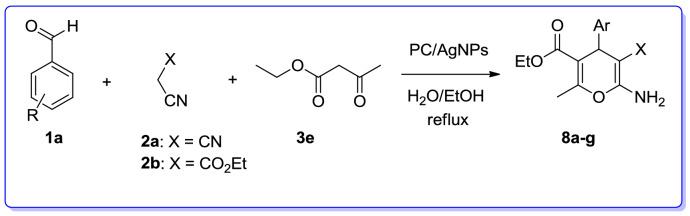

Entry	Ar	X	Time (min)	Product	Yield (%)	m.p. (ºC) Obs	m.p. (ºC) Lit
1	C_6_H_5_	CN	42	**8a**	78	195–197	195–196^65^
2	4-Cl–C_6_H_4_	CN	20	**8b**	87	175–177	170–172^146^
3	4-OMe–C_6_H_4_	CN	22	**8c**	84	142–144	142–144^147^
4	4-NO_2_–C_6_H_4_	CN	17	**8d**	86	177–178	176–178^146^
5	4-OH–C_6_H_4_	CN	40	**8e**	83	176–177	175–177^147^
6	4-Me–C_6_H_4_	CN	25	**8f**	84	177–178	177–179^65^
7	3-NO_2_–C_6_H_4_	CN	23	**8g**	80	171–173	171–173^146^

**Scheme 2:**
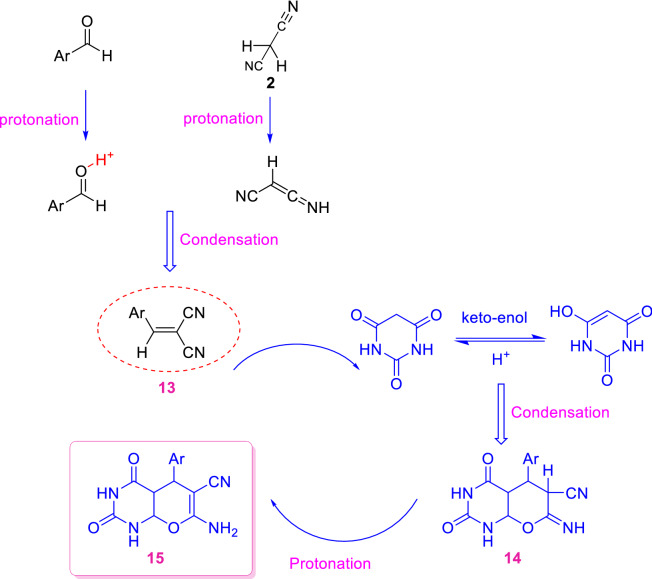
Suggested mechanism for the construction of pyrano[*2,3-d*] pyrimidinones.

A reasonable mechanism for the synthesis of pyrano[*2,3-d*] pyrimidinones was proposed as depicted in Scheme 2. It is presumed that, at first the PC/Ag NPs activates the carbonyl group of the aromatic aldehyde by H protonation. Next, the Knoevenagel condensation of activated aromatic aldehyde with malononitrile (**2**) occurs by the loss of one H_2_O molecule forming arylidenemalononitrile (**13**). In the second step, the nucleophilic (Michael) addition of the enolizable 1,3-dicarbonyl to arylidenemalononitrile generates intermediates (**14**). To end, tautomerization gives the corresponding products (tetrahydrobenzo[*b*]pyrans and pyrano[*2,3-d*]pyrimidinones) (**15**).

In the following, we successfully investigated the catalytic potency of the PC/AgNPs in the synthesis of ethyl 6-amino-5-cyano-2-methyl-4*H*-pyran-3-carboxylates via three-component reaction involving isatin and its derivatives such as isatin and 5-chloro, malononitrile /ethylcyanoacetate and 1,3-dicarbonyl compounds such as dimedone, barbituric acid, ethylacetoacetate, α-naphtol, β-naphtol, 4-hydroxyqumarin and 3-methyl-1*H*-pyrazol-5(4*H*)-one.

To examine the scope and limitation of this catalyzed MCR, isatins such as isatin and 5-chloroisatin, acenaphthenequinone, malononitrile or ethylcyanoacetate and cyclic ketones such as barbituric acid, dimedone, 3-methyl-1*H*-pyrazol-5(4*H*)-one and 4-hydroxycoumarin, acyclic 1,3-dicarbonyl compounds such as ethylacetoacetate, α- naphthol/β-naphthol, 4-hydroxycoumarin, barbituric acids, and 3-methyl-1*H*-pyrazol-5(4*H*)-one were selected for justification of the library of a series of products (Tables 9 and 10). We first investigated the reaction of isatin, and malononitrile with dimedone/ barbituric acid /ethyl acetoacetate or 4-hydroxycoumarin/3-methyl-1*H*-pyrazol-5(4*H*)-one or α-naphtol/β-naphtol. As it was evident from Table 9, this MCR proceeded smoothly, leading to construction of the desired products (**10a–u**) in high to excellent yields in relatively short reaction times.

**Table 9:** Synthesis of spiro-2-amino-4*H*-pyrans (spirochromenes) (**10a**–**u**) in the presence of PC/AgNPs via MCR.
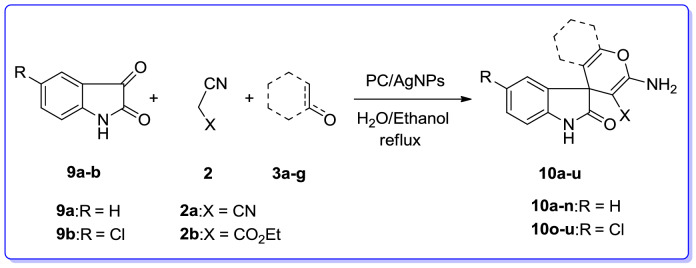

Entry	R	Ar	X	Time (min)	Product	Yield (%)	m.p. (ºC) Obs	m.p. (ºC) Lit
1	H		CN	8	**10a**	95	276–279	278–280^148^
2	H		CN	10	**10b**	96	286–288	286–287^149^
3	H		CN	30	**10c**	89	292–294	292–294^150^
4	H		CN	15	**10d**	96	280–282	281–283^151^
5	H		CN	40	**10e**	89	255–257	255–256^152^
6	H		CN	20	**10f**	87	222–224	220–222^135^
7	H		CN	18	**10g**	78	234–235	233–235^80^
8	H		CO_2_Et	15	**10h**	78%	258–260	260^153^
9	H		CO_2_Et	30	**10i**	93%	268–270	269–271^154^
10	H		CO_2_Et	35	**10j**	78%	252–254	251–253^95^
11	H		CO_2_Et	25	**10k**	85%	284–287	285–287^155^
12	H		CO_2_Et	60	**10l**	73%	174–176	176^153^
13	H		CO_2_Et	40	**10m**	82%	228–230	229^98^
14	Cl		CN	93	**10n**	8	288–290	289–290^156^
15	Cl		CN	96	**10o**	10	292–294	291–293^157^
16	Cl		CN	92	**10p**	18	> 300	> 300^158^
17	Cl		CN	96	**10q**	30	230–232	230–232^158^
18	Cl		CN	91	**10r**	40	263–266	263–265^159^
19	Cl		CN	89	**10s**	20	> 300	> 300^160^
20	Cl		CO_2_Et	89	**10t**	25	269–272	271–272^161^
21	Cl		CO_2_Et	95	**10u**	20	245–247	246–248^158^

**Table 10:** Synthesis of ethyl 6-amino-5-cyano-2-methyl-4*H*-pyran-3-carboxylates (**12a**–**j**) in the presence of PC/AgNPs via MCR.
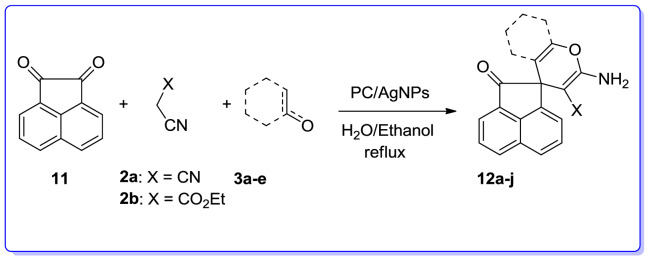

Entry	Ar	X	Time (min)	Product	Yield (%)	m.p. (ºC) Obs	m.p. (ºC) Lit
1		CN	8	**12a**	93	> 300	> 300^135^
2		CN	10	**12b**	94	267–270	268–270^162^
3		CN	15	**12c**	91	291–294	288–292^163^
4		CN	10	**12d**	93	297–299	298–299^164^
5		CN	35	**12e**	85	> 300	> 300^165^
6		CN	25	**12f**	84	> 300	> 300^97^
7		CO_2_Et	20	**12g**	89	> 300	> 300^162^
8		CO_2_Et	25	**12h**	90	261–265	259–262^162^
9		CO_2_Et	35	**12i**	81	238–241	240–242^166^
10		CO_2_Et	20	**12j**	86	246–249	247–248^162^

To further establish the substrate scope of this reaction catalyzed by PC/AgNPs, isatins were employed as substrates and reacted with ethylcyanoacetate and dimedone/ barbituric acid/ethylacetoacetate or 4-hydroxycoumarin/3-methyl-1*H*-pyrazol-5(4*H*)-one or α-naphtol/β-naphtol.

Pleasantly, it was found that the expected products (**10a–u**) were provided in excellent yields. Furthermore, we successfully examined the other derivative of isatin namely 4-chloroisatin.

The three-component reaction of isatin, and malononitrile/ethylcyanoacetate with dimedone/ barbituric acid/ethyl acetoacetate or 4-hydroxycoumarin/3-methyl-1*H*-pyrazol-5(4*H*)-one or α-naphtol/β-naphtol in which gave the expected desired products (**10a–u**) in satisfactory yields (Table 9). Irrespective of the influence of nature of substituent on the isatin, the desired products were provided in high yields. When acenaphthenequinone (**11**) was used the desired spiro-4*H*-pyrans (**12a–j**) were also produced in good yield (Table 10). Noticeably, the reaction with ethylcyanoacetate required longer reaction times than those with malononitrile, which was perhaps because of their lower reactivity (Table 10).

**Scheme 3:**
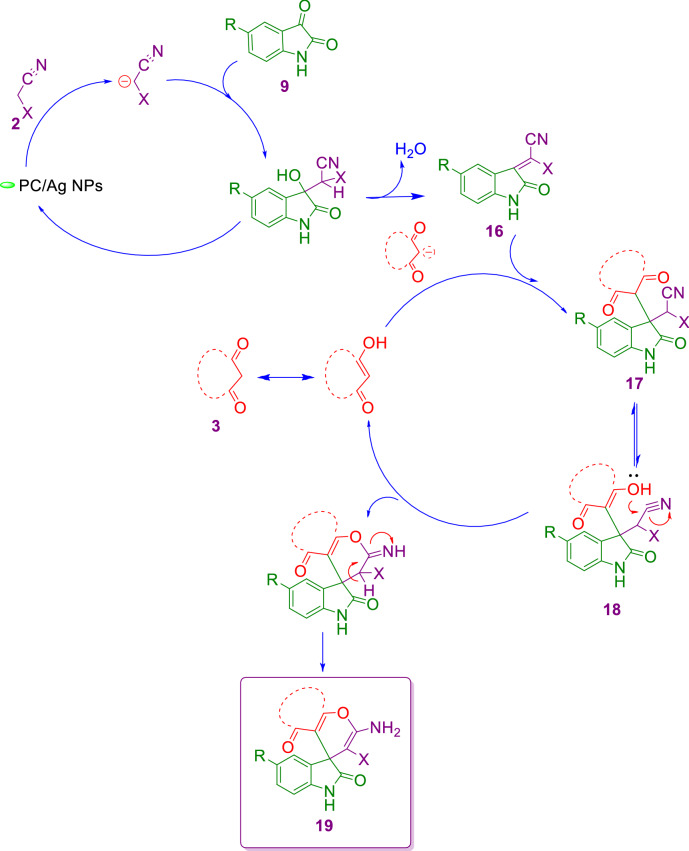
Suggested mechanism for the synthesis of spiro derivatives (**19**).

The process represents a typical sequential cascade reaction in which the isatin (**9**), at first, condenses with malononitrile (**2**) to give isatylidene malononitrile (**16**) in the presence of PC/Ag NPs in refluxing EtOH/water. This step was considered as a rapid Knoevenagel condensation. Next, intermediate (**17**) is attacked via Michael addition of 1,3-dicarbonyl compound (**3**) to afford the intermediate (**18**) with subsequent cycloaddition of hydroxyl group to the cyano moiety to give the desired product (**19**) (Scheme 3). Indeed, the reaction is a cascade reaction via combination of two famous name reactions so called Knoevenagel condensation/Michael addition.

To present the advantages of our novel catalyst, its catalytic activity was compared with the other catalysts reported for the aforementioned MCRs, hitherto (Table 11). The catalytic potency of our novel catalyst (PC/AgNPs) was compared with the same recently reported MCR, involving acenaphthenequinone, malononitrile and dimedone for the preparation of 2′-amino-tetrahydro-2*H*-spiro[acenaphthylene-1,4′-chromene]-3′-carbonitrile (**12b**) with various catalysts such as CaCl_2_,^164^ Fe_3_O_4@_Cs-SO_3_H,^163^ Na_2_EDTA,^165^ HAuCl_4_.3H_2_O,^166^ Meglumine,^135^ Fe_2_O_3,_^167^ HEAA,^168^ Cu(OAc)_2_^.^H_2_O,^169^ Amb-400Cl (IRA-400 Cl),^170^ Fe_3_O_4_@CS-SO_3_H NPs,^171^ C_4_(DABCO-SO_3_H)_2_^.^4Cl,^172^ 1-butyl-3-methylimidazolium hydroxide ([bmim][OH],^173^ Carbon–SO_3_H,^174^ DBU,^175^ (SB-DBU)Cl,^176^ PEG-Ni nanoparticle-catalyzed,^177^ trisodiumcitrate dehydrate,^178^ with that of PC/AgNPs novel catalyst. The results showed that in comparison to other catalysts, our novel catalyst, is superior relative to other tested or reported catalysts from different points of view. Furthermore, our approach gave the desired products in better yield and shorter reaction times. From the green chemistry point of view, the recyclability of the this new nano catalyst as well as employing H_2_O/EtOH as green solvent system render this catalyst green and environmentally benign, favorable for using it in industrial scale (Table 11).

**Table 11:** The comparison of the catalytic activity of PC/AgNPs with previously reported catalysts for the preparation of 2′-amino-tetrahydro-2*H*-spiro[acenaphthylene-1,4′-chromene]-3′-carbonitrile (**12b**).
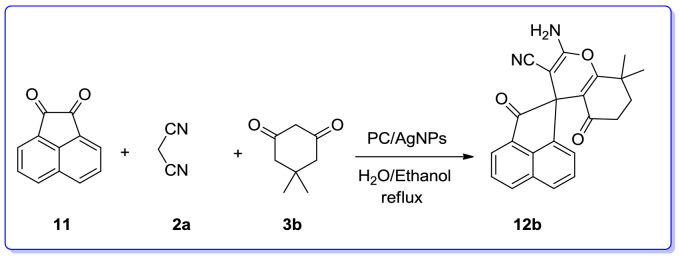

Entry	Catalyst	Catalyst amount (gr)	Time (min)	Temperature	Solvent	Yield (%)	Refs
1	CaCl_2_	0.02	50	r.t	Ultrasonic	96	^164^
2	Fe_3_O_4_@Cs-SO_3_H	0.02	300	Reflux	H_2_O/EtOH	98	^163^
3	Na_2_EDTA	0.01	10	70 °C	Solvent-free	94	^165^
4	HAuCl_4_^.^3H_2_O	0.05	30	70 °C	PEG 400	96	^166^
5	Meglumine	0.05	20	r.t	H_2_O/EtOH	93	^135^
6	Fe_2_O_3_	0.02	240	90 °C	Solvent-free	84	^167^
7	HEAA	0.02	60	90 °C	H_2_O	92	^168^
8	Cu(OAc)_2_.H_2_O	0.02	300	80 °C	Solvent-free	84	^169^
9	Amb-400Cl (IRA-400 Cl)	0.02	10	Reflux	H_2_O	95	^170^
10	Fe_3_O_4_@CS-SO_3_H NPs	0.02	5	r.t	H_2_O/EtOH	92	^171^
11	C_4_(DABCO-SO_3_H)_2_·4Cl	0.01	12	90 °C	H_2_O	98	^172^
12	1-butyl-3-methylimidazolium hydroxide ([bmim][OH		20	r.t	Solvent-free	92	^173^
13	Carbon–SO_3_H	0.01	180	Reflux	EtOH	91	^174^
14	DBU	0.01	15	Reflux	H_2_O	88	^175^
15	(SB-DBU)Cl	0.05	60	r.t	EtOH	98	^176^
16	PEG-Ni nanoparticle	0.0235	10	r.t	PEG	93	^177^
17	trisodium citrate dihydrate	0.01	300	r.t	H_2_O/EtOH	92	^178^
18	PC/AgNPs	0.025	10	Reflux	H_2_O/EtOH	94	This work

### Reusability of catalyst

**Figure 7:**
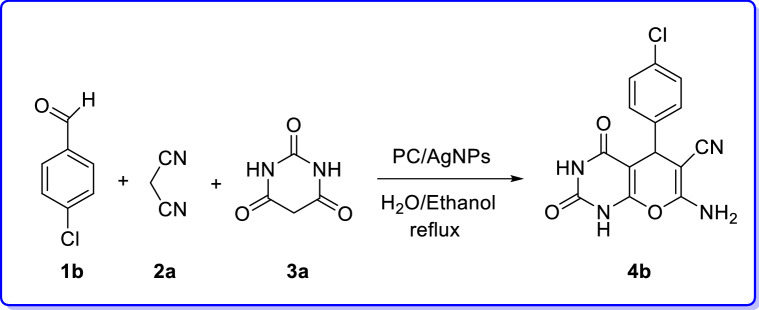
Typical reusability of our new catalyst in the preparation of 7-amino-5-(4-chlorophenyl)-2,4-dioxo-1,3,4,5-tetrahydro-2*H*-pyrano[2,3-*d*]pyrimidine-6-carbonitrile (**4b**).

**Figure 8:**
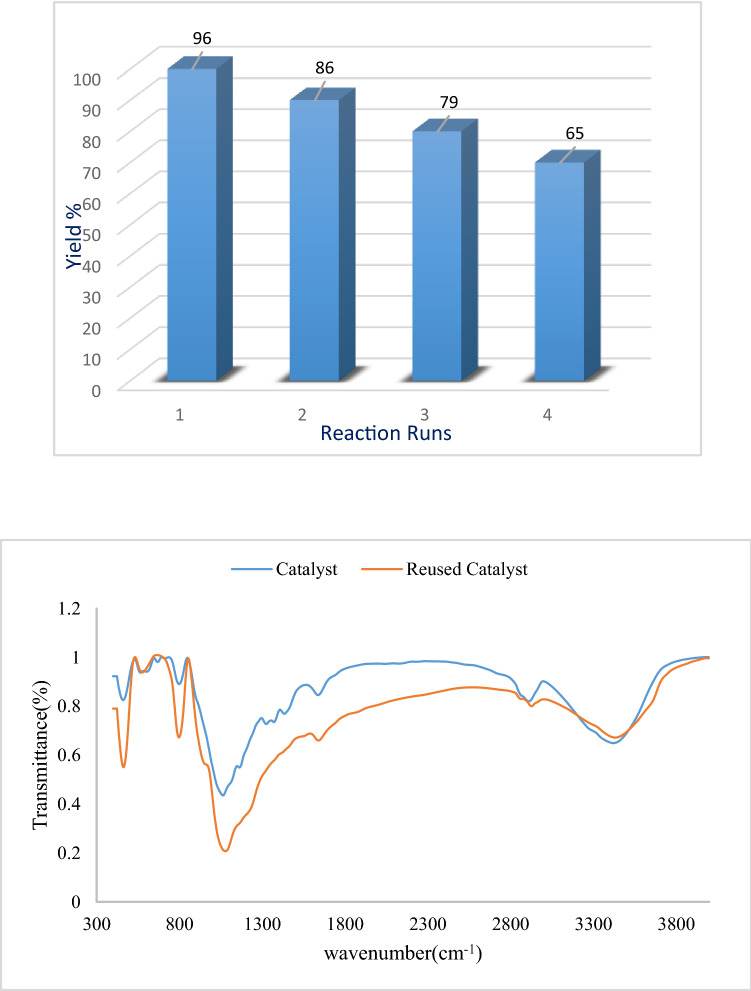
The FT-IR PC/AgNPs and reused catalyst.

The revocability and reusability of the catalyst were also studied. To the purpose the nucleophilic condensation between 4-chlorobenzaldehyde, malononitrile and barbituric acid under optimized conditions was chosen as a model reaction for the preparation of 7-amino-5-(4-chlorophenyl)-2,4-dioxo-1,3,4,5-tetrahydro-2*H*-pyrano[2,3-*d*]pyrimidine-6-carbonitrile (**4b**). After first run, the catalyst was separated by simple filtration under reduced pressure, washed with ethanol. Next, the recovered catalyst was reused in the next run under the same reaction conditions in the model reaction. This investigation showed that the catalyst could be recovered and reused at least three times whiteout significant loss of its catalytic activity (Fig. 7). Worthy to mention that Fig. 8 shows the comparison between the FT-IR PC/AgNPs and reused catalyst.

## Conclusions

In conclusion, the above-presented research opened a gateway to a facile and rapid in situ preparation of Ag nanoparticles immobilized onto the functionalized microcrystalline cellulose/Preyssler heteropolyacid. It was tested as an eco-friendly, benign, and green nanobiocomposite heterogeneous catalyst. The opportunity of collecting three green materials including inorganic polymers, cellulose matrices and nanoparticles can open a green gateway in forthcoming requests such as the design and engineering of green and eco-friendly catalysts based on poly-functionalized polymers. The Taguchi robust design strategy was also employed for the optimization of the experimental parameters for the first time to obtain nanoparticle. The operating factors involved in this process were the concentration of silver nitrate, different loadings PC, pH, and temperature. Absorption spectra of AgNPs showed peak at 440 nm and broadening of peak showed that the particles are poly dispersed. Optimal conditions involved in this study were: performing the reaction in 80 °C, concentration of silver nitrate = 6 mM, pH = 12.5 and loading PC = 1gr. The results obtained under the aforementioned conditions were in good agreement with the data analyzed by Taguchi robust design method. The results of UV–vis absorption undoubtedly confirm the above findings.

Novel PC/AgNPs was successfully employed as catalyst in the synthesis of biologically active 2-amino-4*H*-pyran and spirochromenes. The PC/AgNPs were fully characterization by using standard techniques. The above mentioned catalyst was used in refluxing EtOH/H_2_O providing the corresponding products in good to high yield. The catalyst was recovered and reused for three times without a significant decrease in its efficiency. Other advantages of this catalytic system were that reactions could be performed under mild reaction conditions and in very short reaction times, along with easy product and catalyst separation. This catalyst showed high stability and durability under optimal reaction conditions. The leaching of the AgNPS from the heterogenized catalyst was also found being minimal.

## Supplementary information

Supplementary Information.
