# Supplementary material for: Silver nanoparticles-decorated Preyssler functionalized cellulose biocomposite as a novel and efficient catalyst for the synthesis of 2-amino-4H-pyrans and spirochromenes
Source: Sci Rep. 2020 Sep 3;10:14540. doi: 10.1038/s41598-020-70738-z (PMC7471288; doi:10.1038/s41598-020-70738-z)

**Supplementary for**

**Silver nanoparticles-decorated Preyssler functionalized cellulose biocompositeas: A novel and efficient catalyst for the synthesis of**

**2-amino-4*H*-pyrans and spiro-2-amino-4*H*-pyrans (spirochromenes)**

***via* multicomponent reactions**

Sara Saneinezhad,^a^  Leila mohammadi,^b^ Vahideh Zadsirjan,^a^ Fatemeh F. Bamoharram,*^a^Majid M. Heravi*^b^

*^a^Department of Chemistry, Mashhad Branch, Islamic Azad University, Mashhad, Iran,*

*Email adress: abamoharram@yahoo.com*

*^b^ Department of Chemistry, Alzahra University,Tehran, Iran*

*Email adress: mmheravi@alzahra.ac.ir*

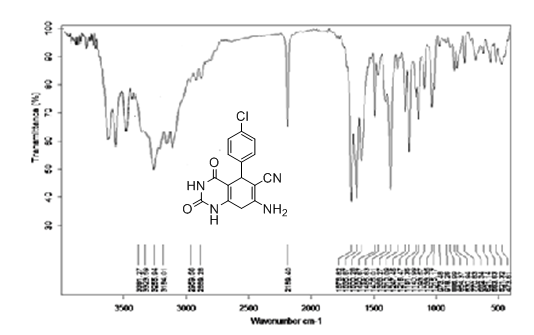


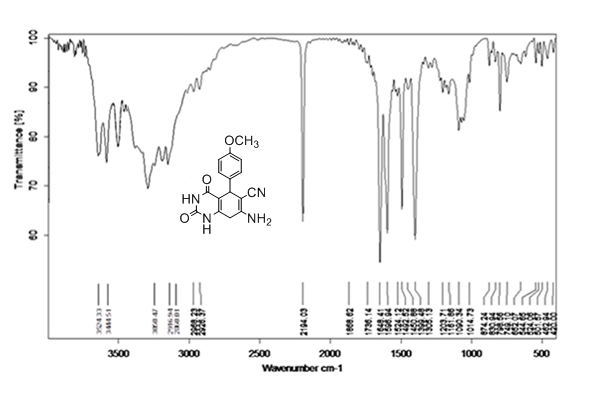


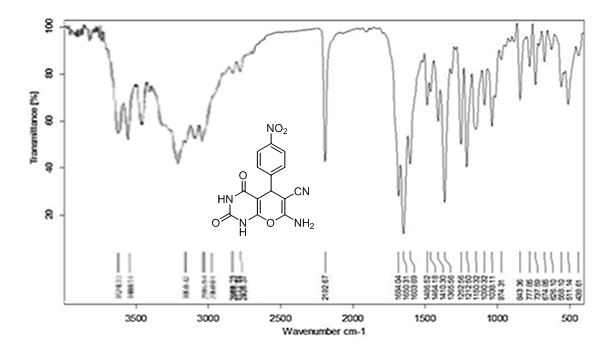


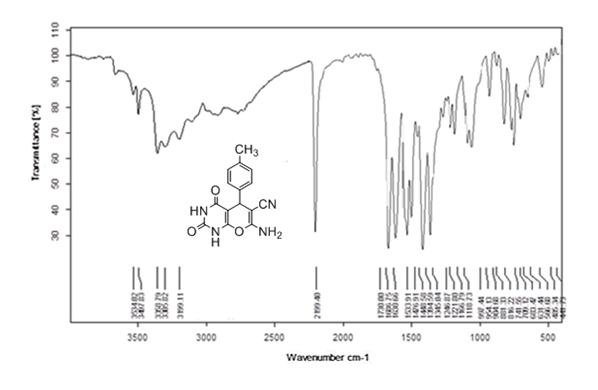


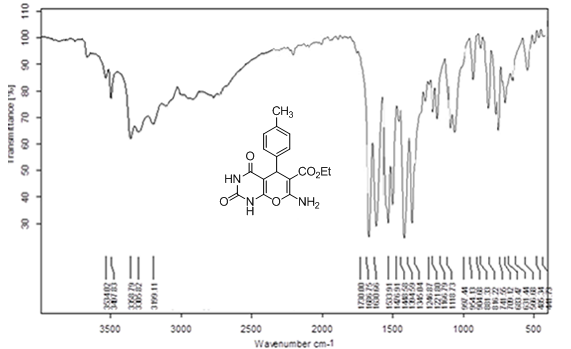


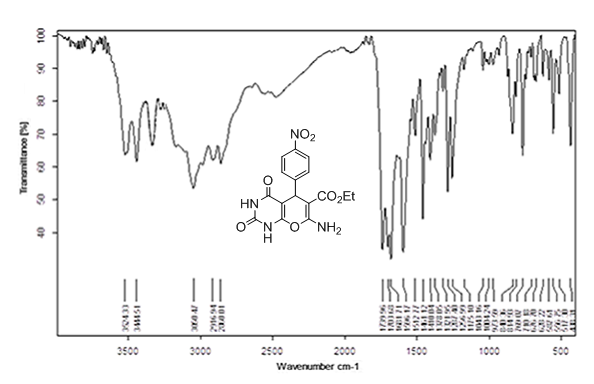


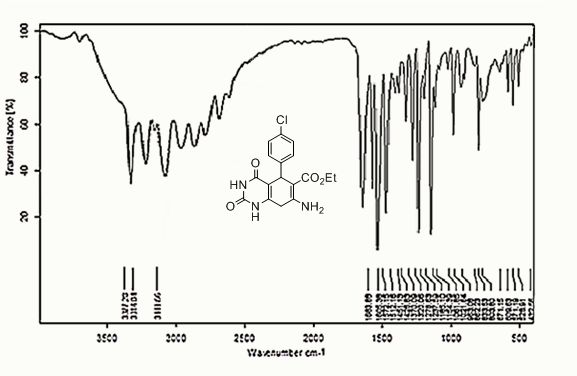


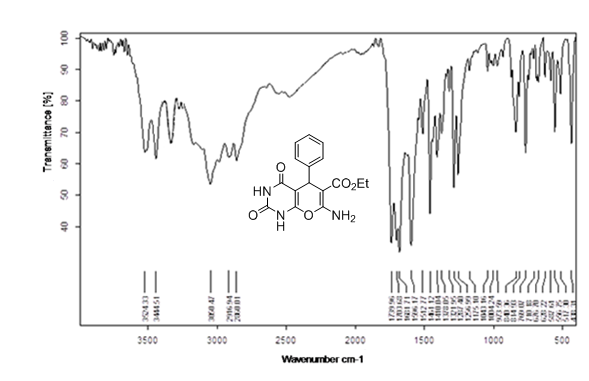


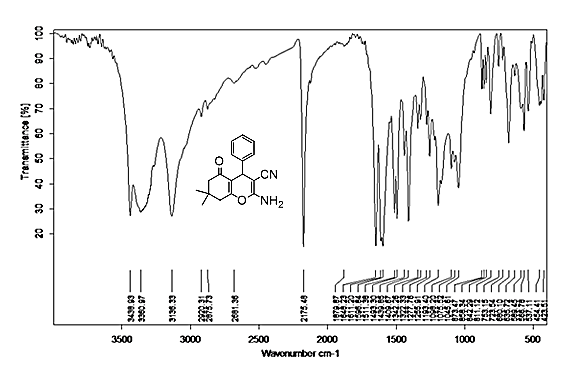


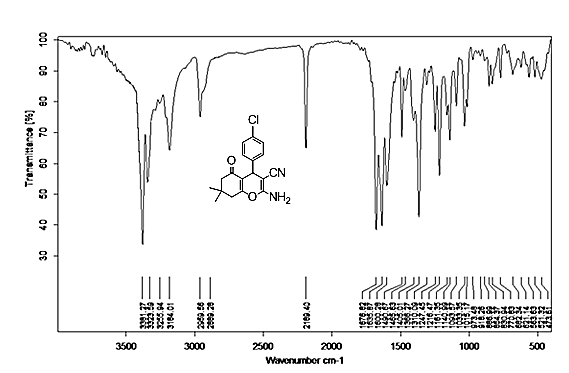


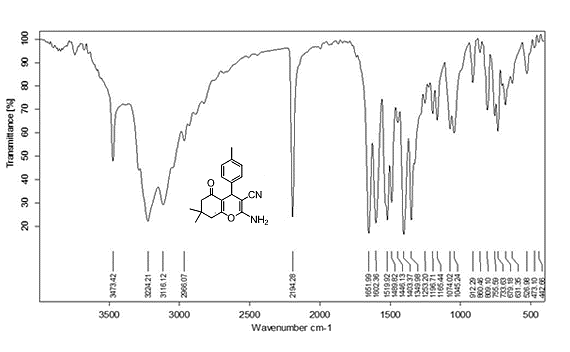


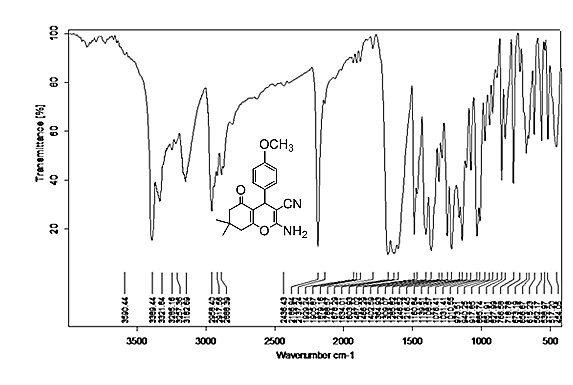


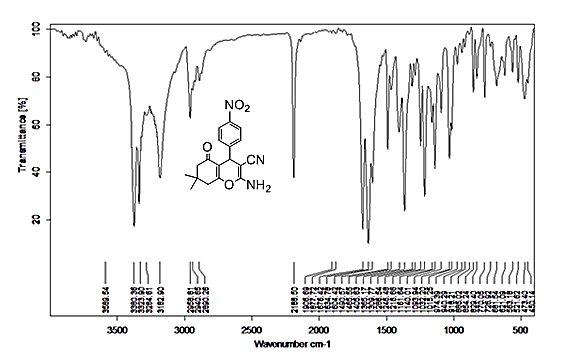


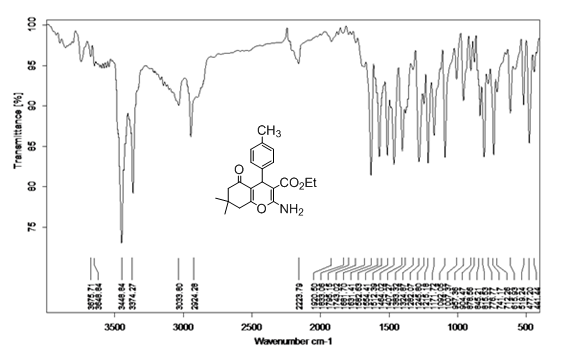


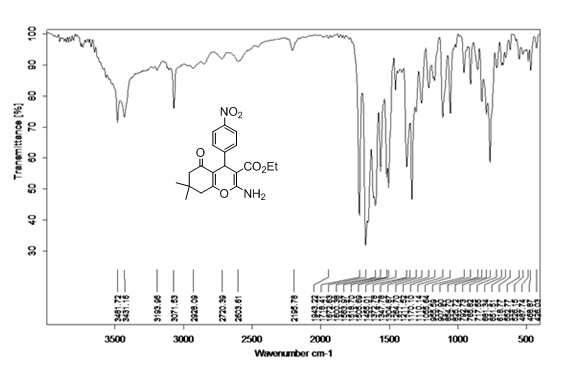


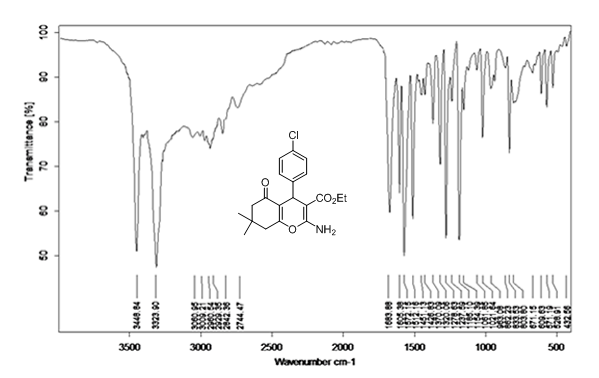


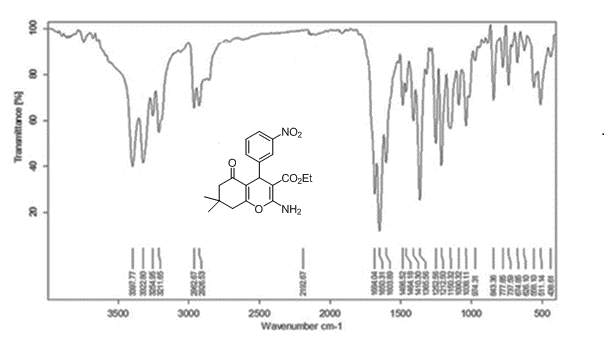


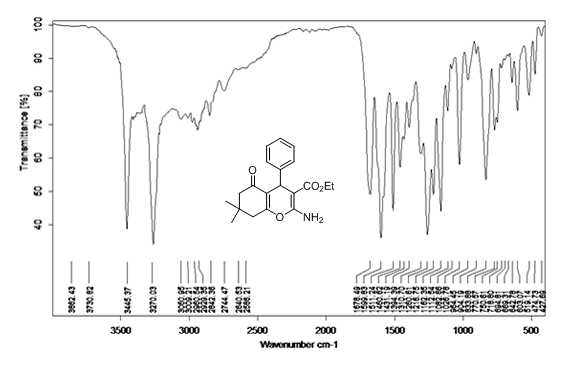


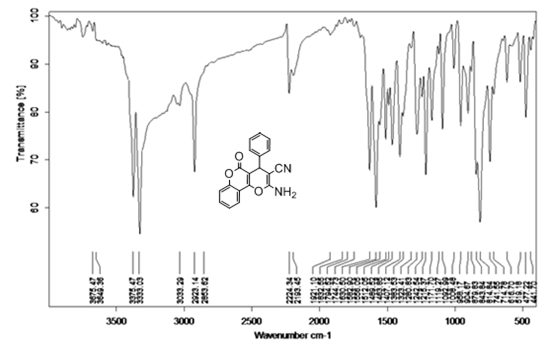


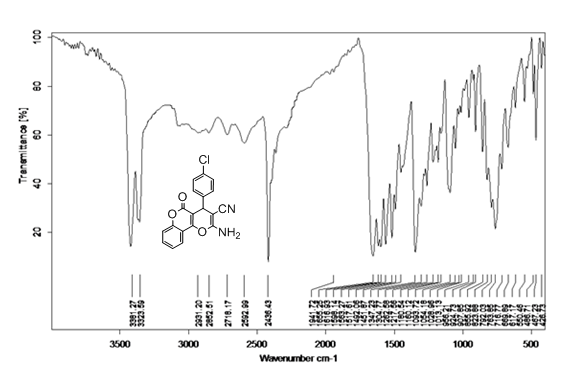


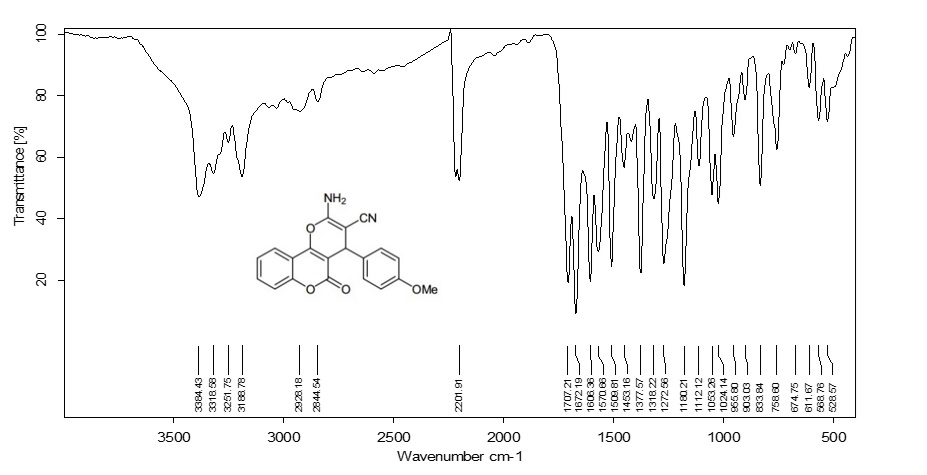


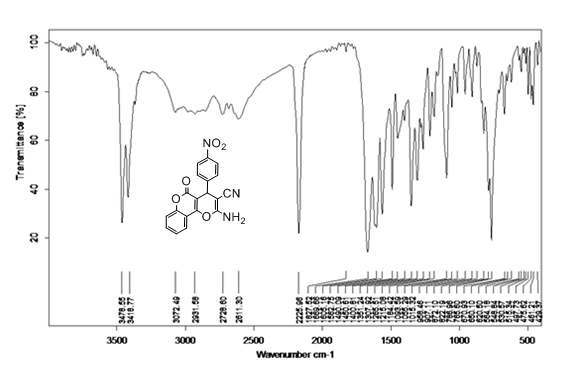


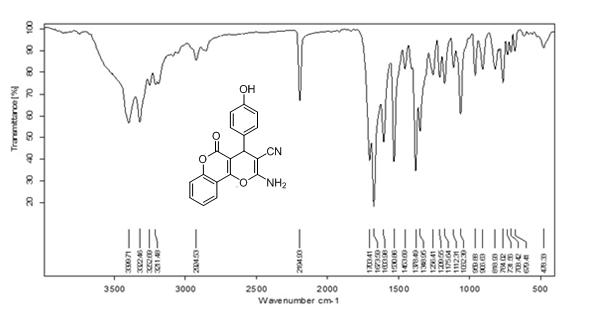


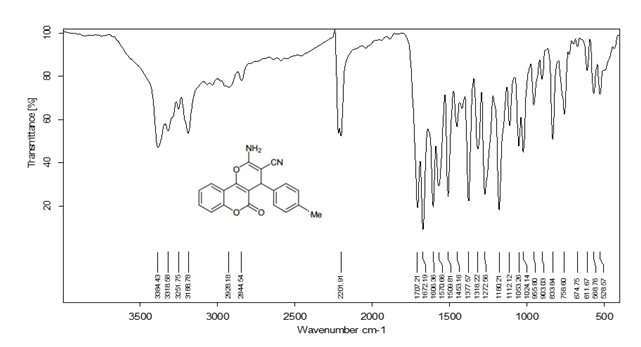


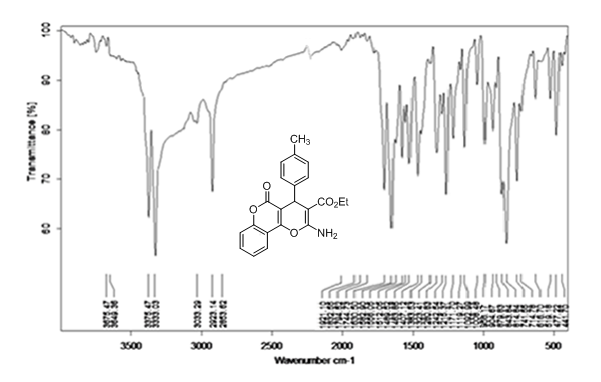


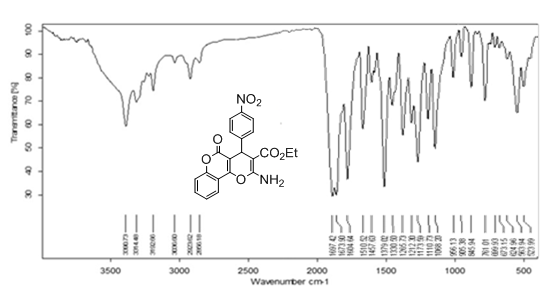

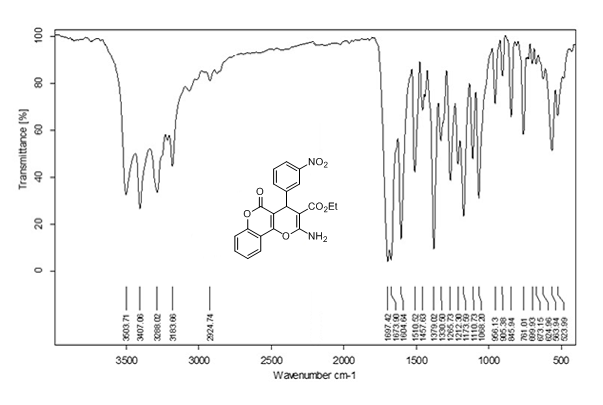


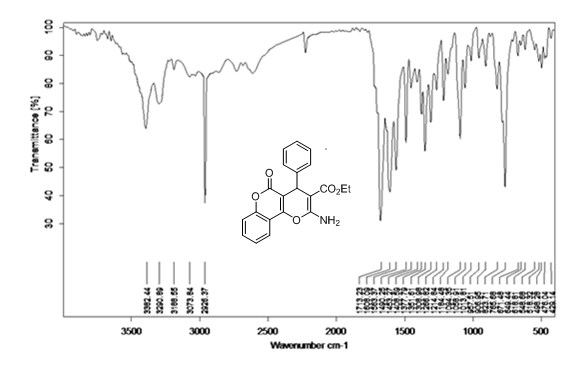


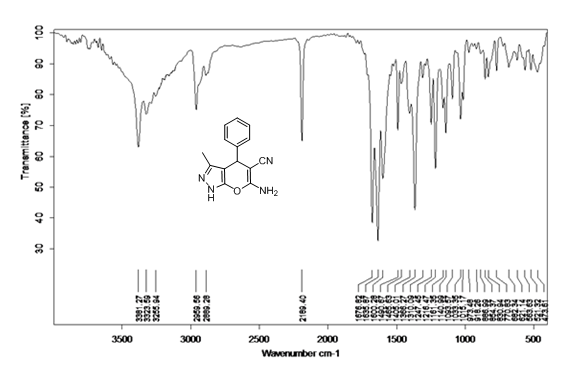


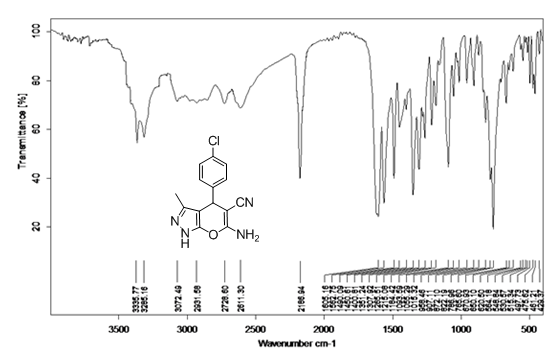


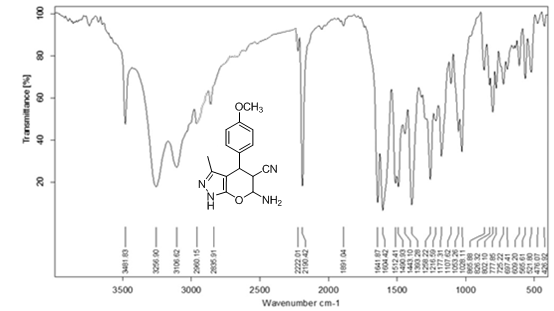


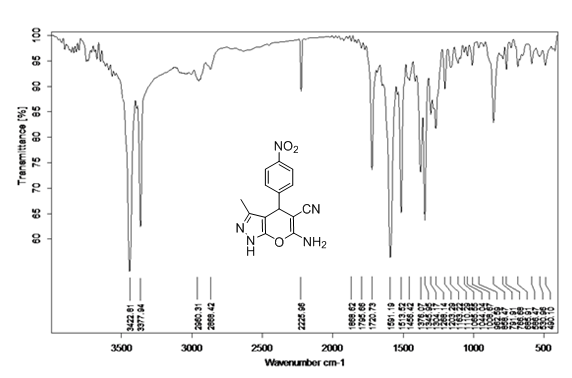

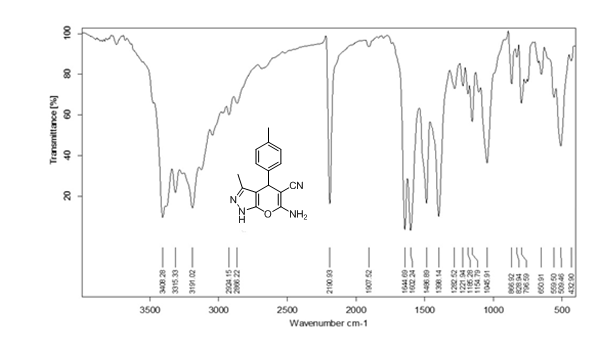

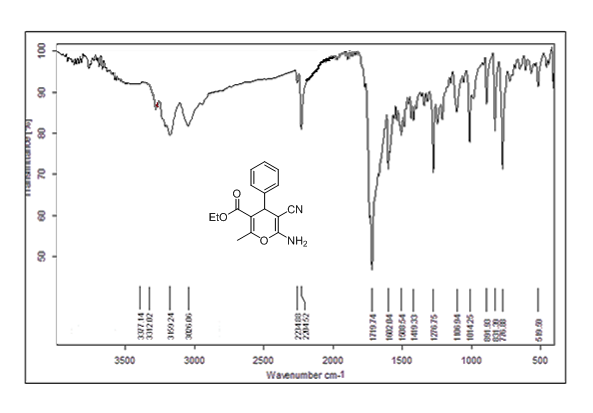


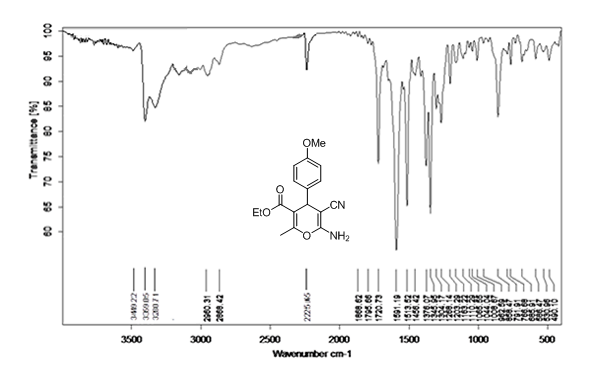


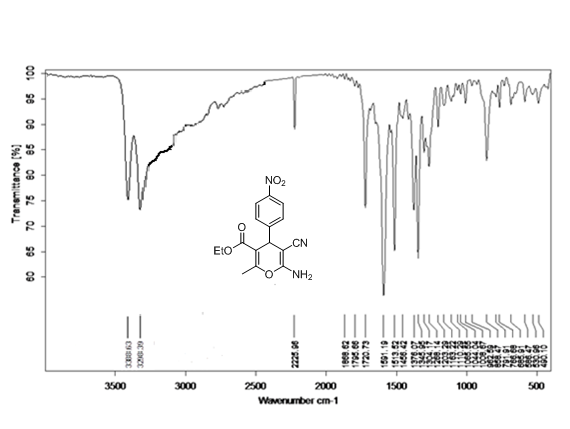


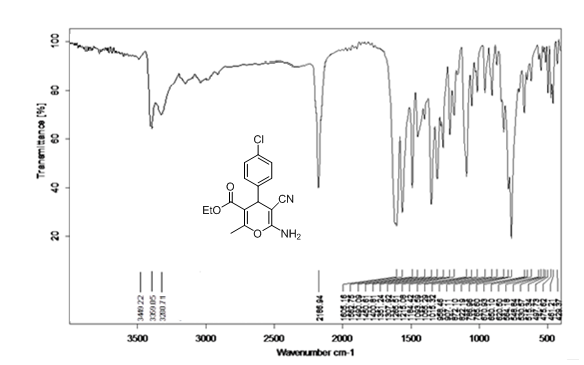


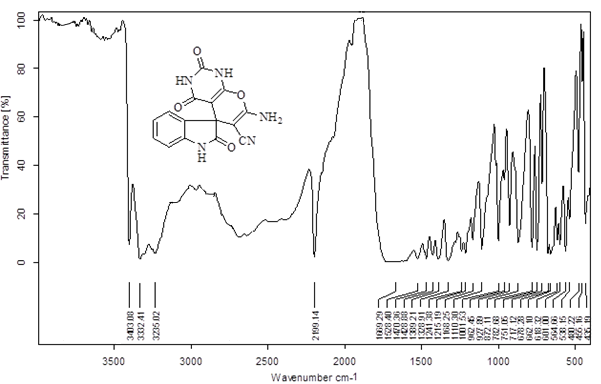


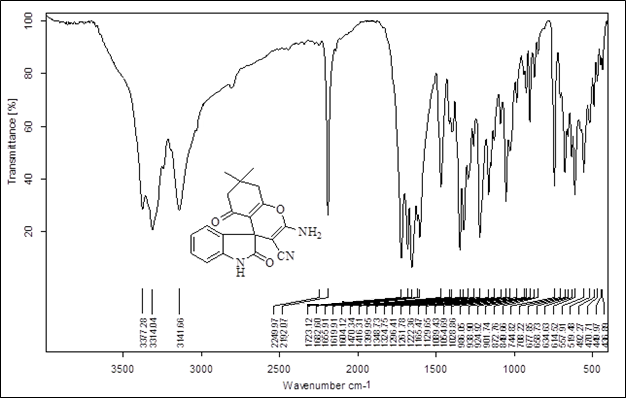


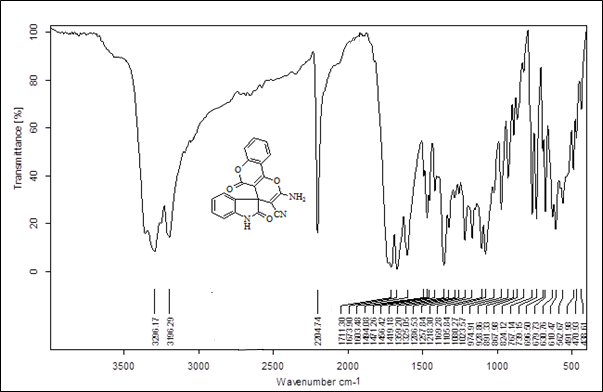


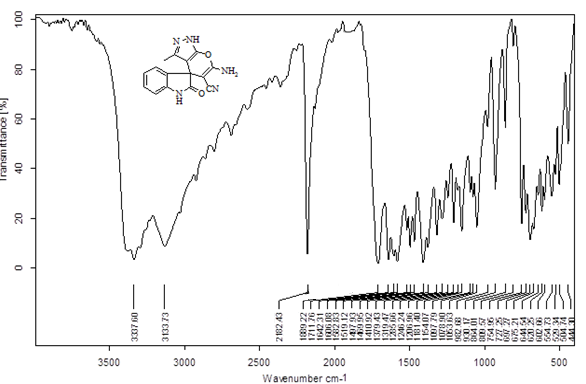


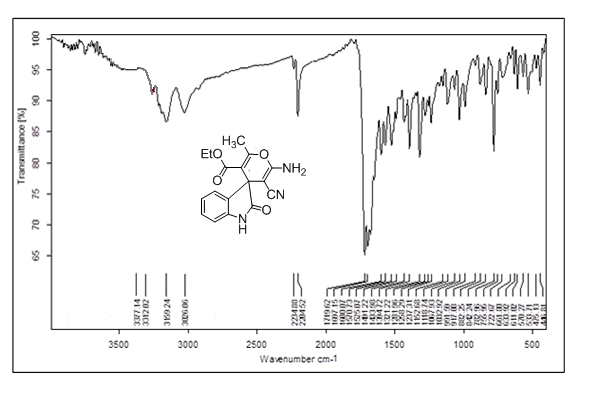


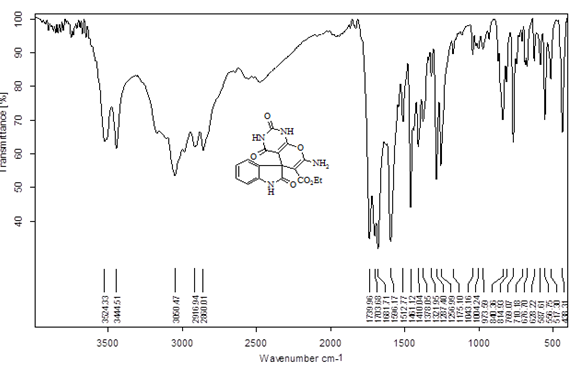


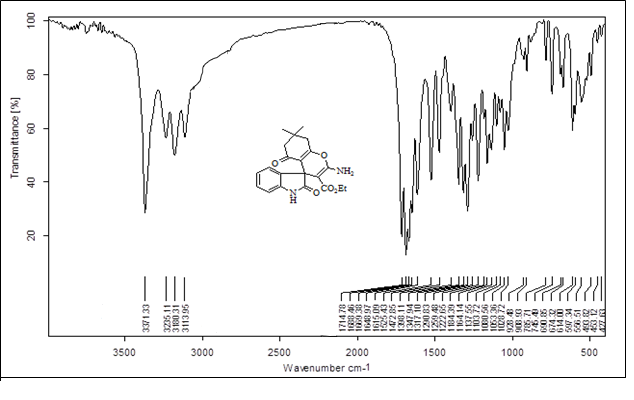


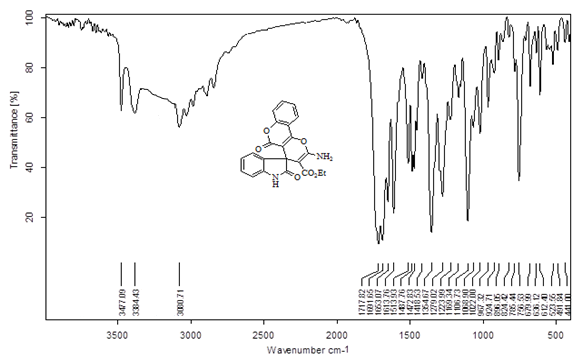

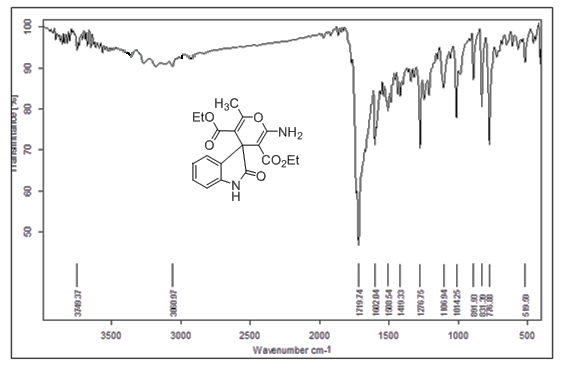


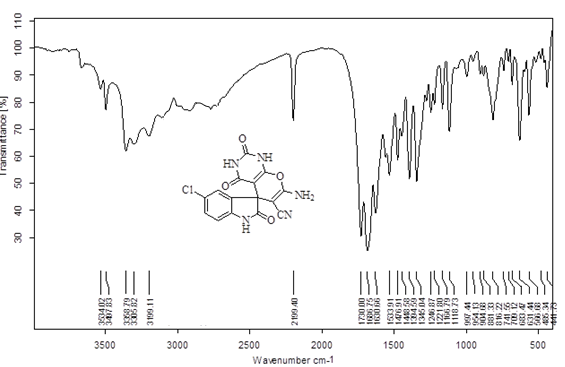


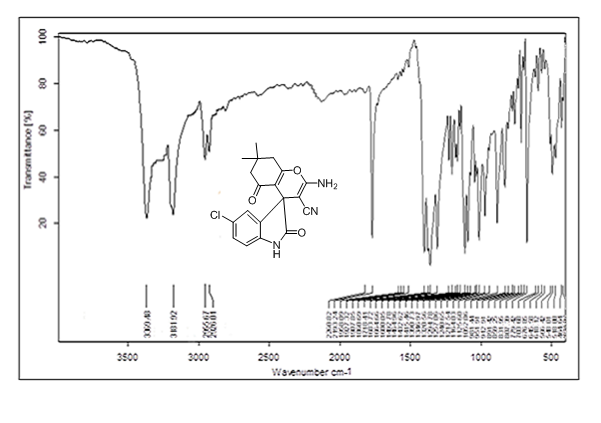

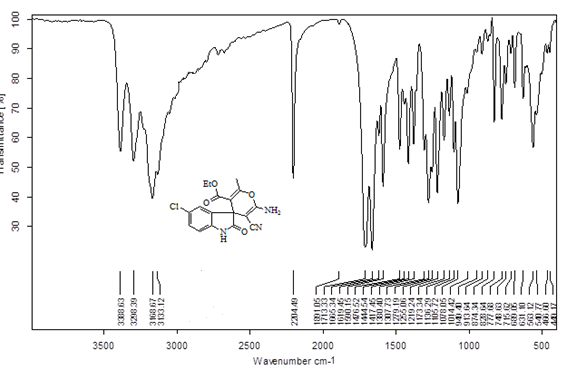


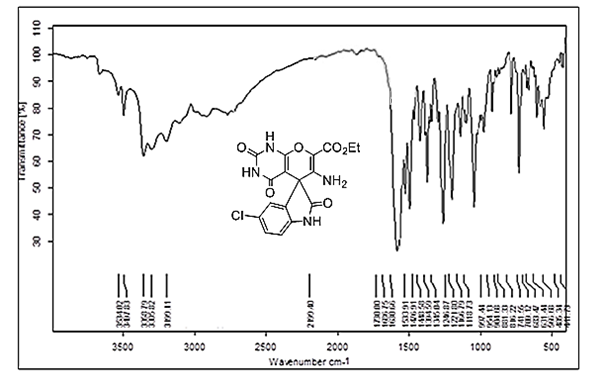

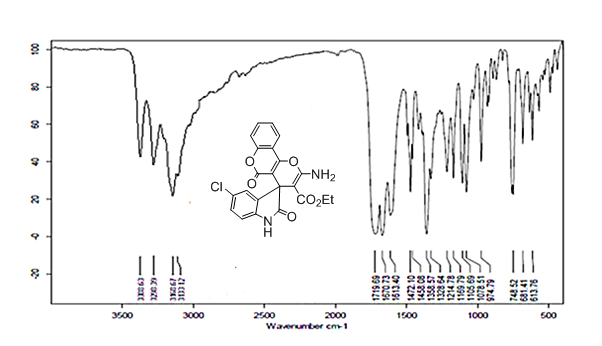

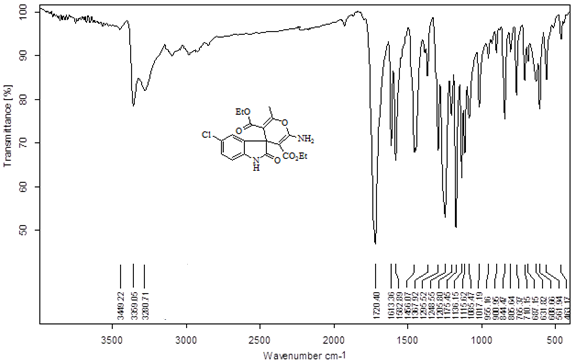


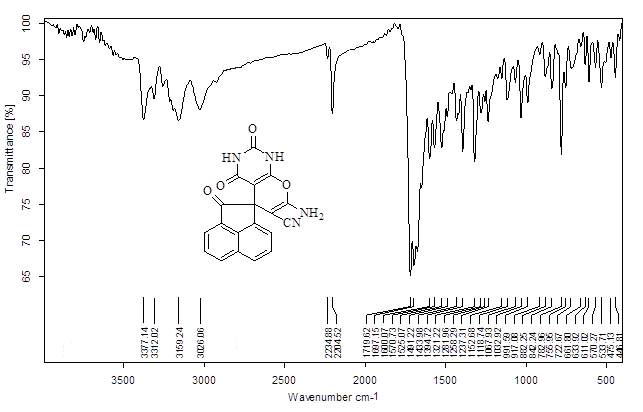


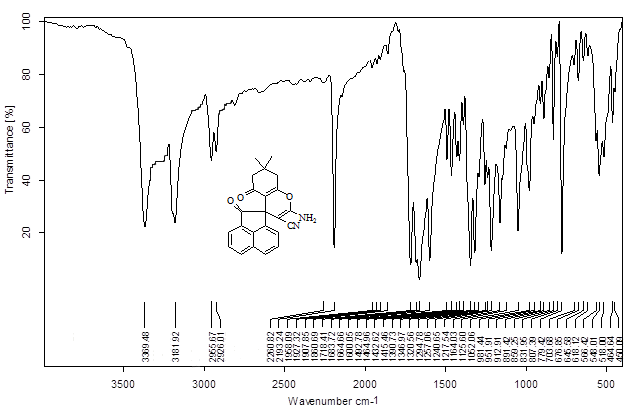


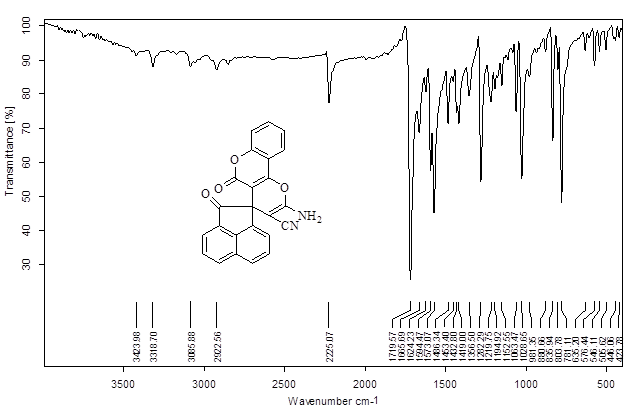


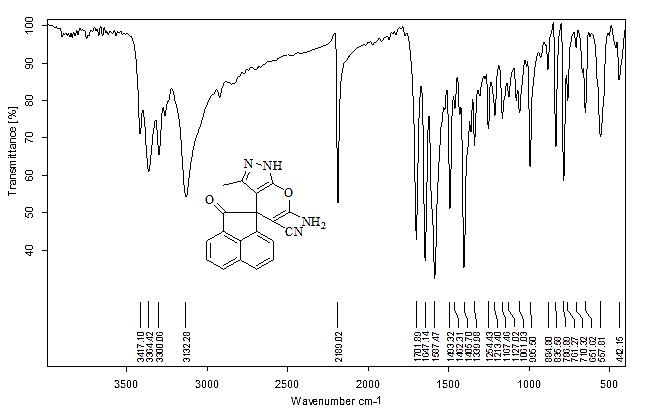


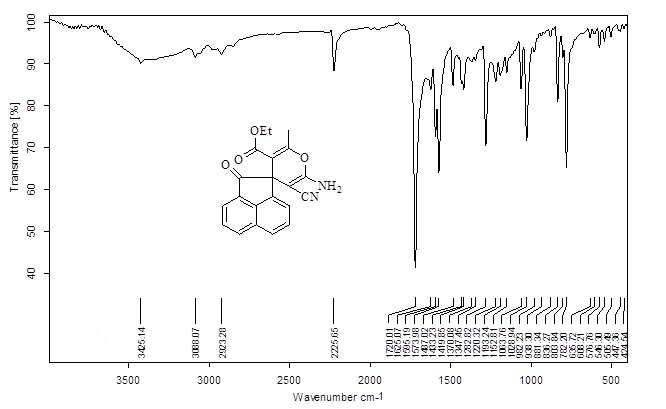


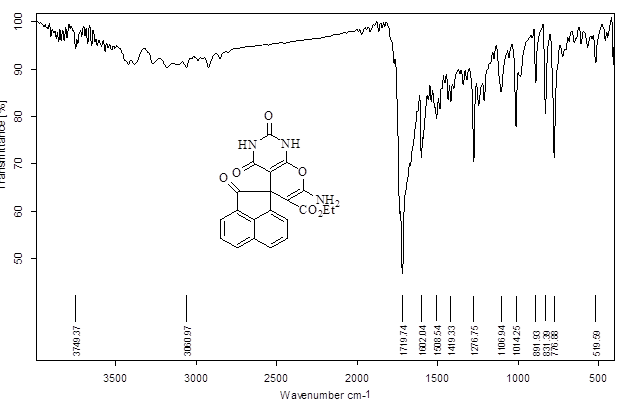


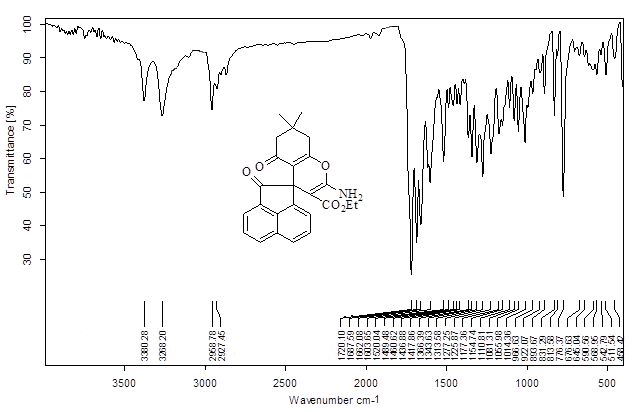


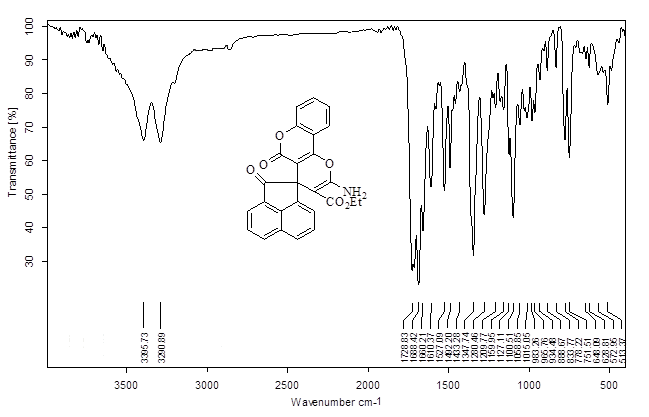


**
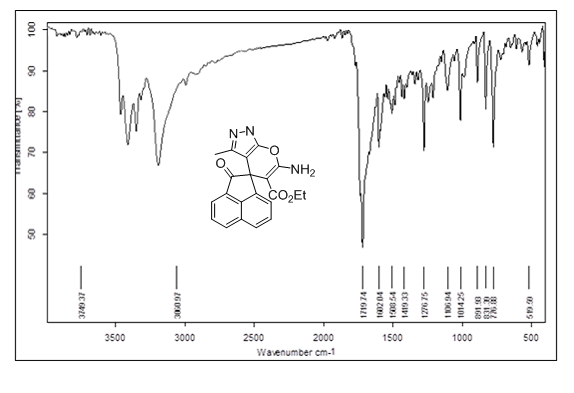
**


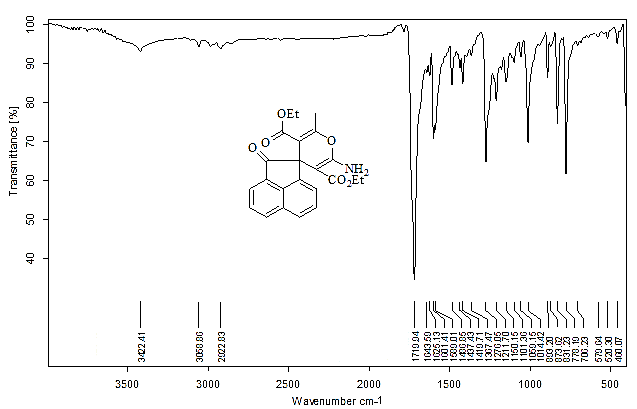


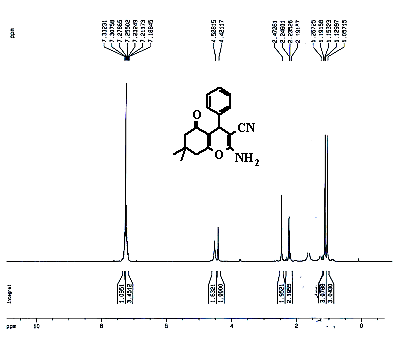


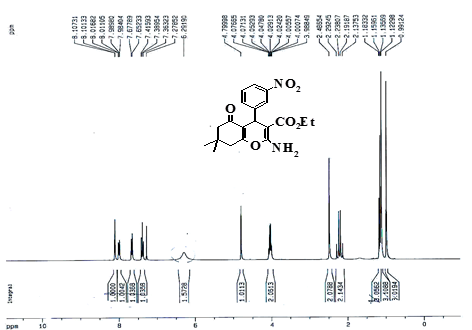


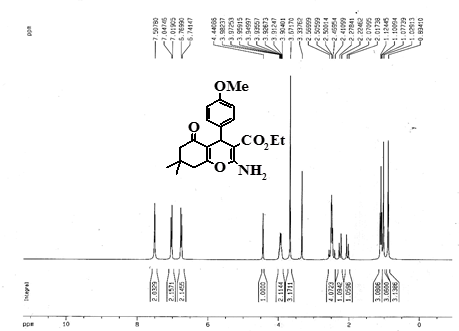

Supplement: Supplementary file 1 — Supplementary Information. [file 41598_2020_70738_MOESM1_ESM.docx]
